# Neuropeptide Y—Graphene Oxide Complexes Inhibit Amygdala NPY‐Receptor Expressing Glutamatergic Pathways and Selectively Remove Aversive Memory In Vivo

**DOI:** 10.1002/advs.76608

**Published:** 2026-07-31

**Authors:** Elisa Pati, Audrey Franceschi Biagioni, Raffaele Casani, Luis M. Arellano, Tommaso Battisti, Gloria Garcia‐Ortega, Neus Lozano, Alberto Bianco, Kostas Kostarelos, Laura Ballerini, Giada Cellot

**Affiliations:** ^1^ International School for Advanced Studies (SISSA/ISAS) Trieste Italy; ^2^ Nanomedicine Lab Catalan Institute of Nanoscience and Nanotechnology (ICN2) CSIC and BIST Campus UAB Barcelona Spain; ^3^ Institute of Neuroscience Universitat Autònoma de Barcelona Barcelona Spain; ^4^ CNRS Immunology Immunopathology and Therapeutic Chemistry UPR3572 University of Strasbourg, ISIS Strasbourg France; ^5^ Centre For Nanotechnology in Medicine Faculty of Biology Medicine & Health The University of Manchester Manchester UK; ^6^ ICREA Barcelona Spain; ^7^ Department of Life Sciences University of Trieste Trieste Italy

**Keywords:** fear memory modulation, graphene oxide nanosheets, nanoplatform engineering, neuronal circuit‐specific targeting, peptide‐functionalized nanomaterials

## Abstract

Therapeutic needs to modulate brain circuits highlight graphene‐based materials (GBMs) as an emerging tool to engineer specific interventions to treat neuro‐diseases. In this context, graphene oxide (GO) nanosheets offer new drug delivery strategies to reach neural cells and signaling networks selectively. To complex and transport neuropeptide Y (NPY), GO was engineered as GO:NPY, and its activity was investigated in regulating excitatory neurotransmission in NPY‐positive synaptic pathways, when delivered to the amygdala, a structure mediating fear memory responses. First, it was shown that in vitro GO:NPY specifically and selectively inhibited glutamate release and suppressed synaptic enhancement via NPY receptors. In a rat model of anxiety disorder, when injected into the amygdala, GO:NPY suppressed contextual fear memory responses via activation of NPY receptors in specific synaptic pathways. This easy‐to‐tune GBM‐based nanoplatforms promise advances in co‐delivery vectors preserving the synaptic specificity needed for treating specific pathological conditions.

## Graphical abstract

A graphene oxide nanocarrier presents neuropeptide Y specifically to NPY receptor‐expressing pathways in the amygdala, whose activation selectively suppresses aversive memory formation, without modulating anxiety responses. This circuit‐restricted drug release strategy may impact future design of neuropsychiatric therapies with limited off‐target effects. Animal images were generated using ChatGPT (OpenAI).

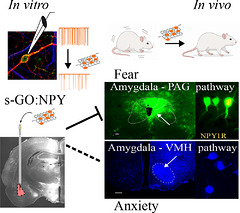

## 1 Introduction

The therapeutic efficacy of drug delivery by vector‐mediated transport relies on strategies enabling cell‐specificity to prevent off‐target effects and unwanted toxicities [1]. In this context, a successful approach is based on the loading of molecules onto drug delivery carriers, such as peptides [2], that can transport or present the vector system toward the desired cell/tissue, specifically recognizing receptors expressed predominantly at the target delivery site [3]. In the manufacturing of such transport systems, nanotechnology plays a fundamental role and allows the design of smart platforms [4]. Graphene‐based materials (GBMs) have attracted considerable interest as the archetypal 2D nanostructures [5, 6], that due to their unique physical‐chemical properties, can be used as carriers for bioactive molecules [7, 8], including neuroscience applications [9, 10]. Among GBMs, graphene oxide (GO) nanosheets have shown potential as a delivery platform [11], thanks to the combination of its nanoscale dimension, enabling interaction with nano‐sized subcellular structures of the nervous tissue [12, 13], with large surface area and versatile chemistry, favoring high loading of multiple chemical compounds [14, 15]. In addition, well‐characterized and purified GO nanosheets can be very biocompatible with respect to other GBMs, without eliciting any significant acute inflammatory adverse effects [16, 17, 18] and can be degraded by immune cells [19, 20] following in vivo administration.

Previous studies reported the use of GO nanosheets for peptide‐based drug delivery to the central nervous system (CNS) [21, 22], however the selectivity of these systems in reaching CNS targets to modulate specific CNS synaptic pathways remains unexplored. In this work, we addressed this challenge by developing a non‐covalent complex between thin graphene oxide nanosheets (s‐GO) and neuropeptide‐Y (NPY). Medical‐grade GO nanosheets with lateral dimensions below 1 µm (s‐GO) [23], previously studied in vitro and in vivo for its nervous tissue biocompatibility [24, 25, 26, 27, 28, 29] was complexed with the neuropeptide Y (NPY), an endogenous modulator peptide of neuronal transmission [30, 31, 32] recently proposed as a therapeutic to cure anxiety disorders [33].

We investigated the synaptic effect of the complex by using in vitro electrophysiological recordings in neuronal cultures, and we found that the s‐GO:NPY complex prevented the induction of chemical long‐term potentiation (cLTP) of excitatory neurotransmission, a cellular model used to probe mechanisms of synaptic plasticity [29]. Then, we tested the bioactivity of s‐GO:NPY in vivo by administering it to a rodent model of post‐traumatic stress disorder (PTSD), exhibiting distinct fear and anxiety responses due to disordered LTP of excitatory synapses in specific pathways of the lateral amygdala (LA) [34, 35].ii Our studies revealed that, when s‐GO:NPY was injected in the LA of PTSD related rats, the complex, similarly to free NPY, reduced fear but not anxiety related behaviors, by reaching and exerting its pharmacological activity specifically on NPY receptors expressing amygdala neuronal circuits, responsible for fear memory responses [36]. Both in vitro and in vivo experiments supported that s‐GO:NPY exerted neuronal modulation via the specific and selective activation of NPY receptors.

## 2 Results

### 2.1 Engineering of Non‐Covalent s‐GO:NPY Complex

The starting materials used for the formation of non‐covalent complexes were the medical‐grade, endotoxin‐free, small (below 1 µm) and thin s‐GO nanosheets, synthesized and characterized extensively as previously described [23], combined with the commercially available 36‐amino acid neuropeptide Y (Figure S1A,B).

**FIGURE 1:**
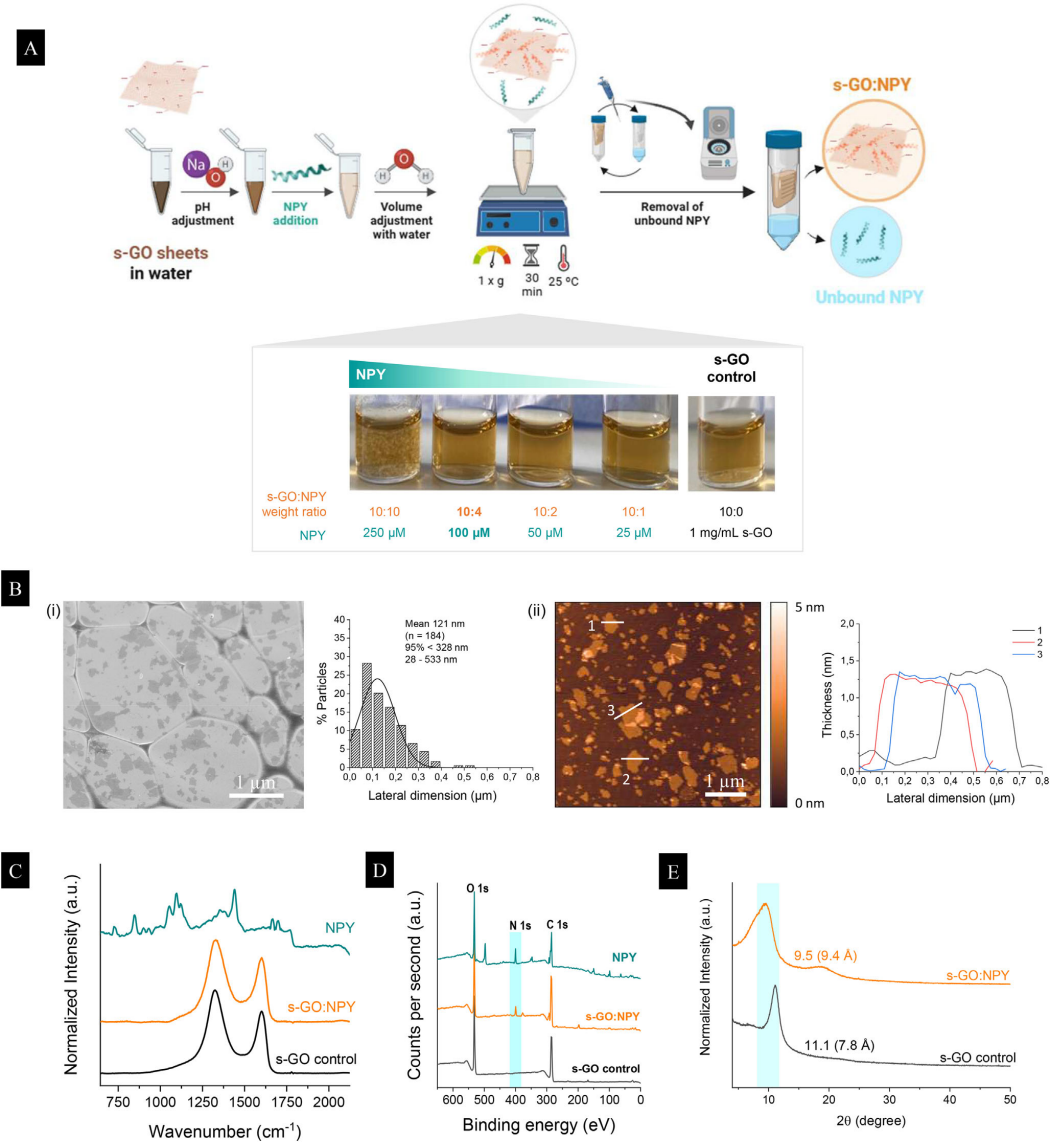
Non‐covalent complexation of s‐GO with NPY. (A) Schematic protocol for the preparation of complexes and pictures of the different s‐GO:NPY mass ratios prepared before the removal of the unbound NPY. s‐GO control corresponds to the processed s‐GO sheets in water after pH adjustment and volume adjustment with water. (B) Morphological characterization of the complex s‐GO:NPY complexes by (i) SEM micrograph with the corresponding lateral dimensions and (ii) AFM height image with the corresponding cross‐section analysis for thickness evaluation. (C) Raman spectroscopy. (D) XPS survey spectra for the structural and elemental composition. (E) XRD curves. Schematic protocol was created with BioRender.com.

Non‐covalent complexes between s‐GO and NPY were prepared by pH adjustment to neutrality of the s‐GO sheets in water and sequential addition of NPY (Figure 1A). Different mass ratios between s‐GO and NPY at 10:10, 10:4, 10:2, 10:1, were used at the same s‐GO concentration of 1 mg/mL. The s‐GO:NPY complex at 10:10 was colloidally unstable immediately after mixing both components, while the complexes at 10:4 and lower mass ratios, were visually stable. Therefore, the non‐covalent s‐GO:NPY complex at 10:4 was selected and fully characterized after following the removal of the unbound NPY by undertaking 4 cycles of centrifugation using ultracentrifugation columns of 100 KDa MWCO. For the quantification of s‐GO sheets and NPY, the individual controls were initially tested to validate the purification method, using UV–vis spectrophotometry for s‐GO and HPLC for NPY, respectively (Figure S2A). s‐GO remains in the concentrate (top fraction), while unbound NPY is passing through the membrane to the filtrates (bottom fraction) (Figure 1A). When loading s‐GO:NPY to the centrifugal columns, the purified s‐GO:NPY complex in the concentrate was evaluated by UV–vis, and almost 100% of starting s‐GO was recovered (Figure S2Bi). In addition, NPY was not detected in the filtrate fraction using HPLC (Figure S2Bii). For further confirmation, a direct NPY quantification method was tested using the SDS/Nu Page kit (Figure S2Biii), obtaining comparable results as the indirect method previously described.

Morphological characterization of the s‐GO:NPY complexes was studied by scanning electron microscopy (SEM) and atomic force microscopy (AFM) (Figure 1B). Lateral dimension distribution was extracted from an SEM micrograph, with 95% of the sheets below 328 nm and a mean peak centered at 121 nm; while the thickness from AFM heigh images showed values between 1 and 2 nm (1 – 2 sheets). The analysis revealed the preservation of discrete s‐GO flakes without significant alteration to their lateral dimensions and thickness after NPY complexation. Additional spectroscopic techniques were used to further validate the NPY complexation onto the s‐GO surface. FTIR spectra of s‐GO and s‐GO:NPY (Figure S3) show no shifts or new bands in the C = O, C–O, and O–H regions, indicating no clear evidence of extensive covalent bonding between GO and NPY under the studied conditions. Raman spectroscopy (Figure 1C) showed the retention of the graphitic lattice after NPY complexation. The appearance of the nitrogen contribution N1s at 400 eV in x‐ray photoemission spectroscopy (XPS) (Figure 1D) after NPY complexation, confirmed the presence of NPY onto the s‐GO surface. The x‐ray diffraction (XRD) spectra (Figure 1E) for the s‐GO:NPY complex, the characteristic 2θ value for s‐GO of 11.1°, shifts to a lower value of 9.5°, indicating an increased basal spacing attributed to the intercalation of the peptide.

The colloidal stability of the s‐GO:NPY complex was assessed using dynamic light scattering (DLS) and zeta potential measurements (Figure S4A–C). Sizes of 200 nm, polydispersity index of 0.3, and zeta potential values of −50 mV remained constant for over 60 days, comparable to the s‐GO control. Moreover, the absence of NPY detachment from the s‐GO sheets was assessed over 42 days by HPLC (Figure S4D) upon storage protected from light at room temperature.

Furthermore, XPS analysis of s‐GO after 30 days of storage provided clear evidence of the persistent association of NPY with the s‐GO surface. The survey spectra and high‐resolution N 1s spectra of the aged complex revealed the continued presence of nitrogen‐containing species attributable to NPY (Figure S4E,F). Consistent with these findings, direct quantification of NPY forcedly detached from a sample stored for 2 years showed no detectable loss of peptide loading compared with freshly prepared s‐GO samples and free NPY controls (Figure S4G,H). These results demonstrate the remarkable long‐term stability of the s‐GO complex under storage conditions.

These findings substantiate the formation of a robust and stable non‐covalent s‐GO:NPY complex, in addition to the absence of endotoxin content assessed by the TNF‐α expression test (Figure S5), rendering it suitability for further biological studies.

### 2.2 NPY Complexed to s‐GO Reduces Glutamatergic Transmission via Activation of NPY Receptors

**FIGURE 2:**
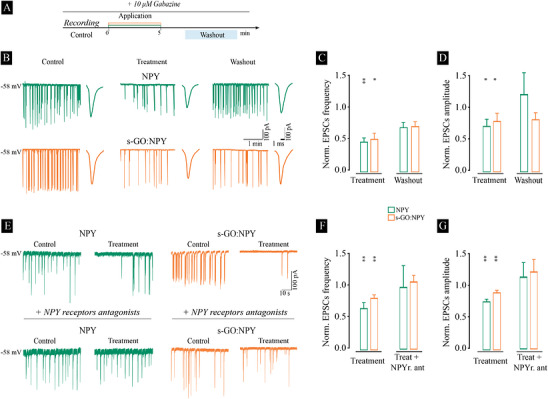
s‐GO:NPY reduces glutamatergic transmission in vitro via NPY receptors activation. (A) Sketch of the experimental setting. Neurons were recorded with a patch‐clamp pipette before the treatments, during the 5 min application of s‐GO:NPY or NPY, and after their washout (in light blue), while excitatory synaptic activity was isolated in 10 µm of gabazine. (B) Exemplificative traces of recordings for the different treatments (NPY in green and s‐GO:NPY in orange), showing as NPY, free or in complex with s‐GO induced a transient decrease in neuronal activity after peptide application, an effect that was recovered upon washout. On the right of each trace, averaged EPSCs measured in the different phases of the experiment are reported for the two treatments. Bar plots of normalized EPSCs frequency (C) and amplitude (D) for the two treatments during the different experimental phases. (E) Representative traces of recordings performed during the application of NPY, both in the complex (orange traces, right) or free (green traces, left), and in the absence (top traces) or presence (bottom traces) of Y1 and Y2 receptor antagonists BIBO3304 and BIIE0246 (30 nm each). No modification of NPY‐driven synaptic activity was observed in the presence of NPY receptor antagonists. Bar plots of normalized EPSCs frequency (F) and amplitude (G) for the two treatments in the presence or absence of NPY receptor antagonists. ^**^ *p* <0.01; ^*^ *p* <0.05.

The biological activity of NPY in specifically modulating excitatory synaptic transmission when delivered after complexation onto the s‐GO nanoplatform was studied using single‐cell patch‐clamp recordings. The synaptic activity in dissociated hippocampal cultures, where the formation of functional glutamatergic synapses and the expression of NPY receptors have been previously reported [37]. Individual pyramidal cells were voltage‐clamped and excitatory postsynaptic currents (EPSCs) detected in the presence of GABA_A_ receptor‐blocker gabazine (10 µm). We compared NPY (1 µm) modulation of EPSCs with that induced by s‐GO:NPY (10 µg/mL and 1 µm, nanomaterial and peptide, respectively), both applied (5 min, sketched in Figure 2A) [30, 38, 39] via the extracellular saline solution.

Representative recordings in Figure 2B show control EPSCs (left column) represented by inward currents of variable frequencies (2.39 ± 0.54 Hz) and amplitudes (346 ± 95 pA, *n* = 17 cells); on the right of each recording, the average EPSC traces are shown, displaying similar kinetic values (Table S1). In cells where NPY (in green) or s‐GO:NPY (in orange) were applied, a down regulation of the excitatory synaptic activity was observed, while no changes were detected when control saline solution was similarly applied (Figure S6). EPSCs kinetics did not change among all treatments (Table S1), however glutamatergic events’ frequencies and amplitudes (normalized to controls; see electrophysiology methods) were reduced by both NPY and s‐GO:NPY applications, as summarized by pooled data in the bar plots of Figure 2C,D. Normalized EPSCs frequencies were significantly reduced by NPY to 0.49 ± 0.08 (*n* = 7 cells) and similarly by s‐GO:NPY to 0.56 ± 0.11 (*n* = 10 cells), in respect to control values (*p* <0.01 for NPY and *p* <0.05 for s‐GO:NPY; Figure 2C). For both these treatments, washout partially recovered EPSCs frequency to control levels (0.75 ± 0.07 in NPY and 0.75 ± 0.11 in s‐GO:NPY; both *p* >0.05; Figure 2B,C).

NPY and s‐GO:NPY depressed EPSC amplitudes respect to controls (0.71 ± 0.11 in NPY and 0.80 ± 0.13 in s‐GO:NPY; *p* <0.05 for NPY and s‐GO:NPY; Figure 1B,D), also this effect was reversed after washout in both cases (1.23 ± 0.32 in NPY and 0.79 ± 0.12 in s‐GO:NPY, both *p* >0.05, Figure 2B,D).

In a different set of experiments, we tested the specificity, i.e. via NPY receptor activation, of NPY and s‐GO:NPY synaptic modulation. As depicted in Figure 2E, we applied free NPY (green traces, left) or complexed to s‐GO (orange traces, right) in the absence (top traces) or presence (bottom traces) of Y1 and Y2 receptor antagonists (BIBO3304 and BIIE0246, respectively, 30 nm each) [40, 41]. In both cases, we did not observe any NPY‐driven downregulation of the excitatory synaptic activity in the presence of such antagonists, confirming that also the s‐GO:NPY complex exerted its bioactivity through NPY receptors (in the presence of NPY receptors antagonists, normalized EPSCs frequencies were 1.03 ± 0.37 in NPY and 1.1 ± 0.1 in s‐GO:NPY; normalized EPSC amplitudes were 1.14 ± 0.22 in NPY and 1.28 ± 0.35 s‐GO:NPY, *n* = 4 and *n* = 3 respectively; in the absence of NPY receptors antagonists: normalized EPSCs frequencies were 0.66 ± 0.08 in NPY and 0.82 ± 0.04 in s‐GO:NPY; normalized EPSC amplitudes were 0.77 ± 0.04 in NPY and 0.90 ± 0.02 in s‐GO:NPY, *n* = 11 and *n* = 12 cells respectively; *p* <0.01, Figure 2E–G).

### 2.3 s‐GO:NPY Complex Prevents Synaptic Potentiation of Amygdala Neurons

**FIGURE 3:**
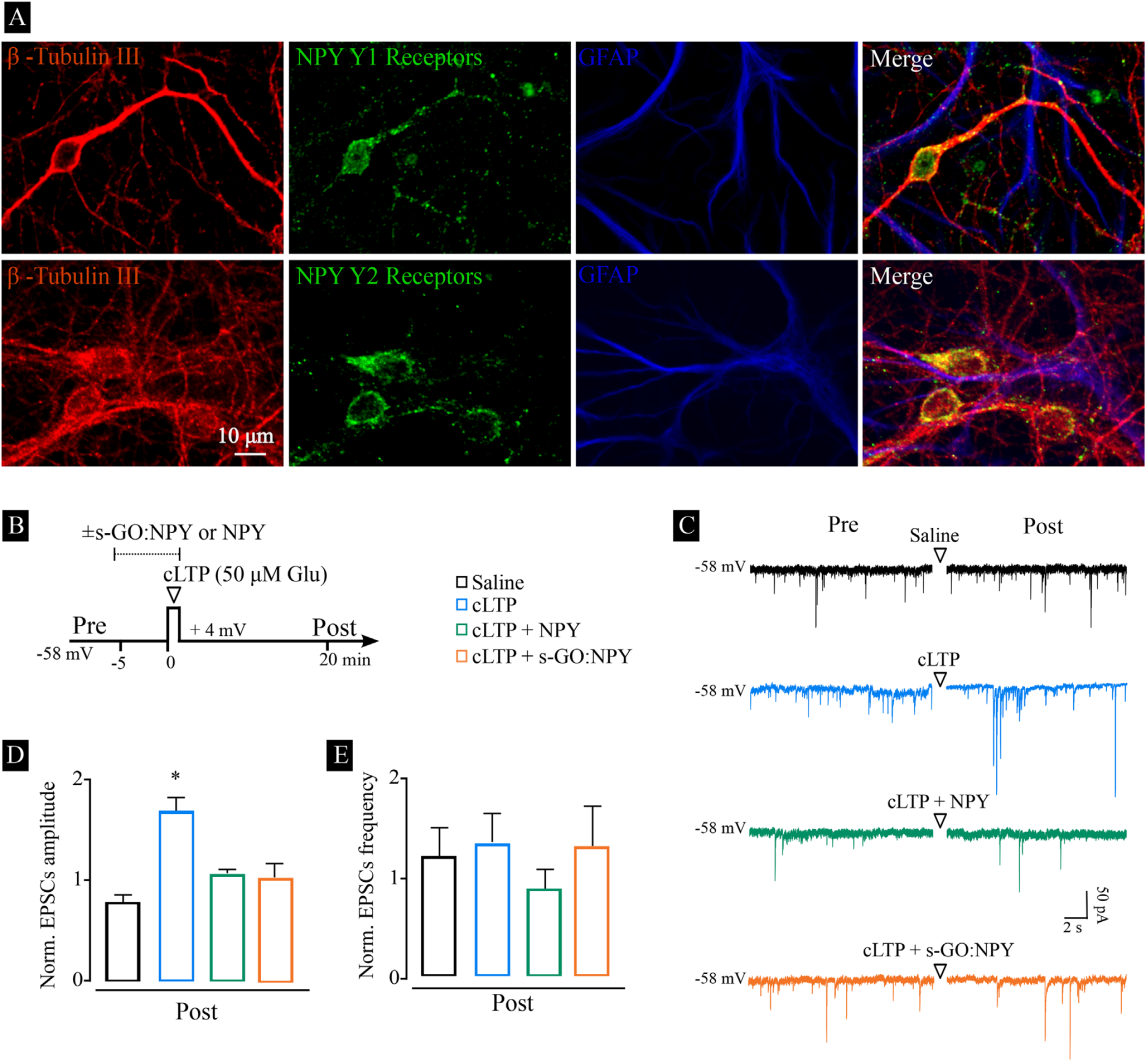
s‐GO:NPY prevents cLTP in dissociated amygdala cultures. (A) Representative fluorescence microscopy images of cultured amygdala cells showing the labelling for β‐tubulin III‐positive neurons (in red) and neuropeptide Y receptors (in green) type 1 (NPY1; top) and type 2 (NPY2; bottom). In blue, GFAP‐positive astrocytes. (B) Sketch of the experimental setting. While recorded with a patch‐clamp pipette in voltage‐clamp mode, neurons were treated with NPY or s‐GO:NPY 5 min before and during the protocol to induce cLTP. This consisted in a 30 s‐long lasting application of glutamate (50 um) during membrane potential depolarization to positive values. (C) Representative traces of recordings performed pre‐ and post‐ treatments. Note that the increment of EPSCs amplitude after cLTP induction (in blue) was prevented by the application of s‐GO:NPY (in orange) or NPY (in green). Bar plots of normalized EPSC amplitudes (D) and frequency (E) measured in a time window 18–24 min post‐cLTP. The application of NPY or s‐GO:NPY co‐applied to cLTP rescued the increased normalized EPSCs amplitudes observed in cLTP‐treated cells, while the EPSC frequency was not affected. No changes in EPSCs amplitude (1.16 ± 0.23) and frequency (0.87 ± 0.09) were found in saline‐treated cells (*n* = 10 cells). ^*^ *p* <0.05.

Since synaptic long‐term potentiation is a phenomenon at the molecular basis of amygdala‐circuits mediated contextual fear memory in vivo [34], we patch‐clamped principal neurons in amygdala dissociated cultures to test in vitro the efficacy of NPY and of s‐GO:NPY in preventing, by down‐regulation of glutamate neurotransmission, synaptic plasticity. We used a paradigm of cLTP to induce in amygdala neurons, expressing both NPY Y1 and Y2 receptors (Figure 3A), stable up‐regulation of excitatory synapses via a brief exposure (30 s) to glutamate (50 µm, sketched in Figure 3B) [28].

We monitored voltage clamped EPSCs (isolated on the basis of their kinetic properties, see methods and [28]) and, as shown in Figure 3C tracings, following cLTP (light blue current traces) neurons displayed an enhanced EPSCs amplitude, with no changes in frequency, respect to baseline; such a change was absent in neurons when exposed (30 s) to extracellular recording solution only (in black). When we applied NPY (1 µm; in green) or s‐GO:NPY (10 µg/mL and 1 µm, respectively; in orange) 5 min before and during the 30 s long‐lasting cLTP induction (sketched in Figure 3B), both the free or complexed peptides prevented cLTP induction and EPSCs amplitude enhancement (Figure 3C). Bar plots from pooled experiments in Figure 3D show the significant increase in EPSCs amplitude (1.63 ± 0.13, *n* = 10 cells; normalized to pre‐treatment, see methods; *p* <0.01) when measured at 18–24 min post cLTP, while it was clearly prevented by NPY or s‐GO:NPY (in NPY 1.07 ± 0.04, *n* = 5 neurons; in s‐GO:NPY 1.03 ± 0.14, *n* = 6 neurons; *p* >0.05 in respect to their pre‐cLTP baseline values). In all the experimental conditions, we never measured significant differences in normalized EPSCs frequencies measured at 18–24 min post cLTP (in cLTP 1.36 ± 0.28; in NPY 0.89 ± 0.19 and in s‐GO:NPY 1.32 ± 0.39 *p* >0.05; Figure 3E). These results suggested that NPY complexed to s‐GO, similarly to the free peptide, prevented synaptic potentiation in amygdala circuits by a downregulation of glutamate neurotransmission.

### 2.4 s‐GO:NPY Injected Into the LA Exclusively Reduces Fear Memory‐Related Behavior

LTP of glutamatergic synapses in the LA circuits in vivo, is responsible for fear conditioning and the development of long‐term pathological behaviors within the framework of anxiety disorders (Pape and Pare, 2011), in particular PTSD [42]. We used a classical contextual fear conditioning paradigm and PTSD model [28, 29] to test the ability of s‐GO:NPY, delivered to the amygdala, to selectively prevent the consolidation of synaptic plasticity in NPY receptor‐positive neurons. In the PTSD model used, a stressor cue (i.e. predator odor) is known to induce LA glutamatergic neurons LTP [28] in the two main neuronal pathways supporting fear and anxiety responses [43, 44, 45], suggested to differ for diverse expression of NPY Y1 receptors, with the former presenting them and the later not [36].

**FIGURE 4:**
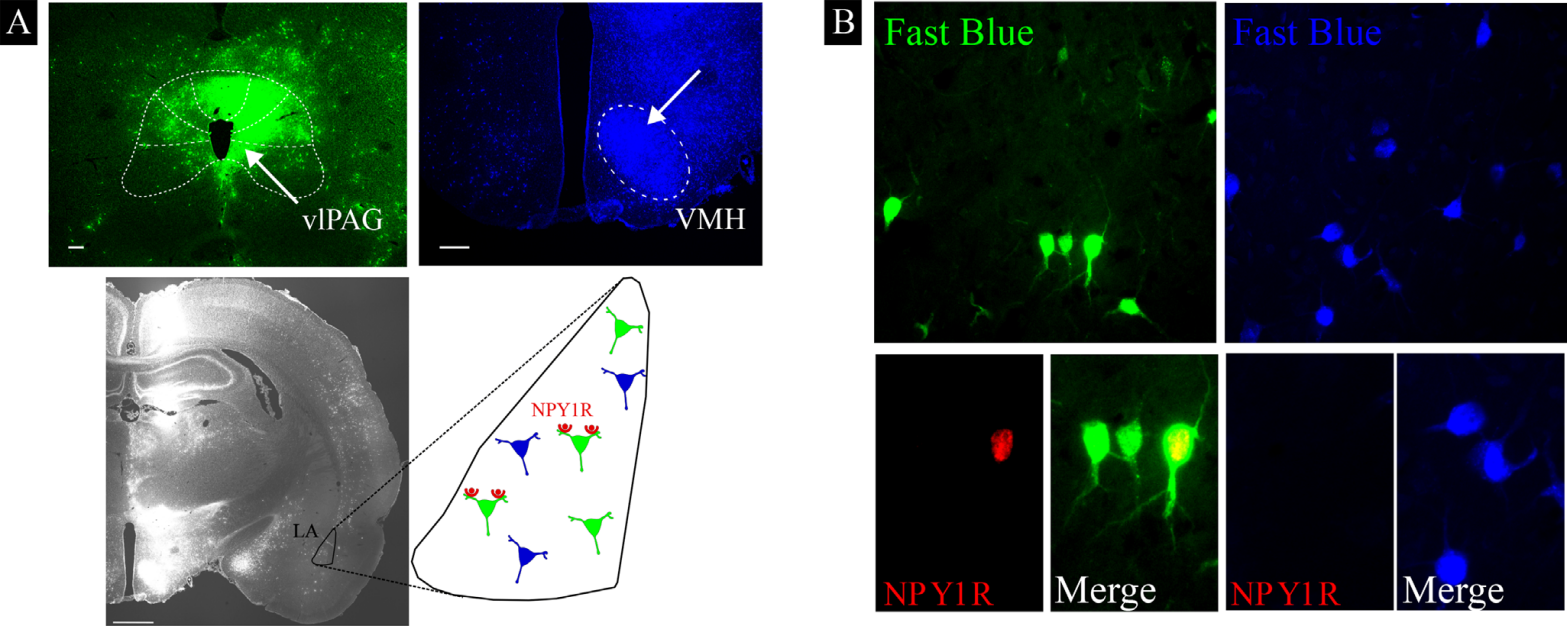
Differential NPY receptors expression in LA‐vlPAG and LA‐VMH pathways. (A) Top, representative photomicrographs of rat brain sections demonstrating the microinjection point of Fast Blue neurotracer into vlPAG and VMH. Bottom, confocal micrograph and schematic diagram illustrate the projection between LA and vlPAG and VMH, demonstrating the exclusive presence of NPY Y1 receptors (NPY1R) on projections innervating the vlPAG. (B) Photomicrograph of a section of the LA showing co‐localization of the projections innervating the vlPAG with NPY1R, while no NPY1R expression was observed in LA projections to VMH (*n* = 2). Scale bars = 100 µm (vlPAG and VMH); 1 mm (brain slice); 25 µm (LA).

First, we confirmed the differential expression of NPY receptors in the two neuronal pathways, by injecting in naïve animals the fluorescent retrograde neurotracer Fast Blue into two CNS regions both innervated by LA neurons, the ventrolateral periaqueductal gray (vlPAG) [46], involved in defensive fear responses, or the ventromedial hypothalamus (VMH), which regulates anxiety (Figure 4A) [47, 48]. When injecting the neurotracer into the VMH, we observed sparse projections in the anterior portion of the LA, while the middle portion exhibited a denser and more robust ipsilateral network of fibers, between bregma coordinates −2.52 and −3.24 mm. Regarding the projections from vlPAG, robust fibers were detected at the LA posterior portion, bregma −3.84, in only one hemisphere projecting from the LA. Additionally, at the middle portion, we observed fewer projections from the LA to the vlPAG. By immunofluorescence, we showed that the fibers traced from vlPAG expressed Y1 NPY receptors, while the fibers from VMH lacked the presence of this receptor (Figure 4B).

**FIGURE 5:**
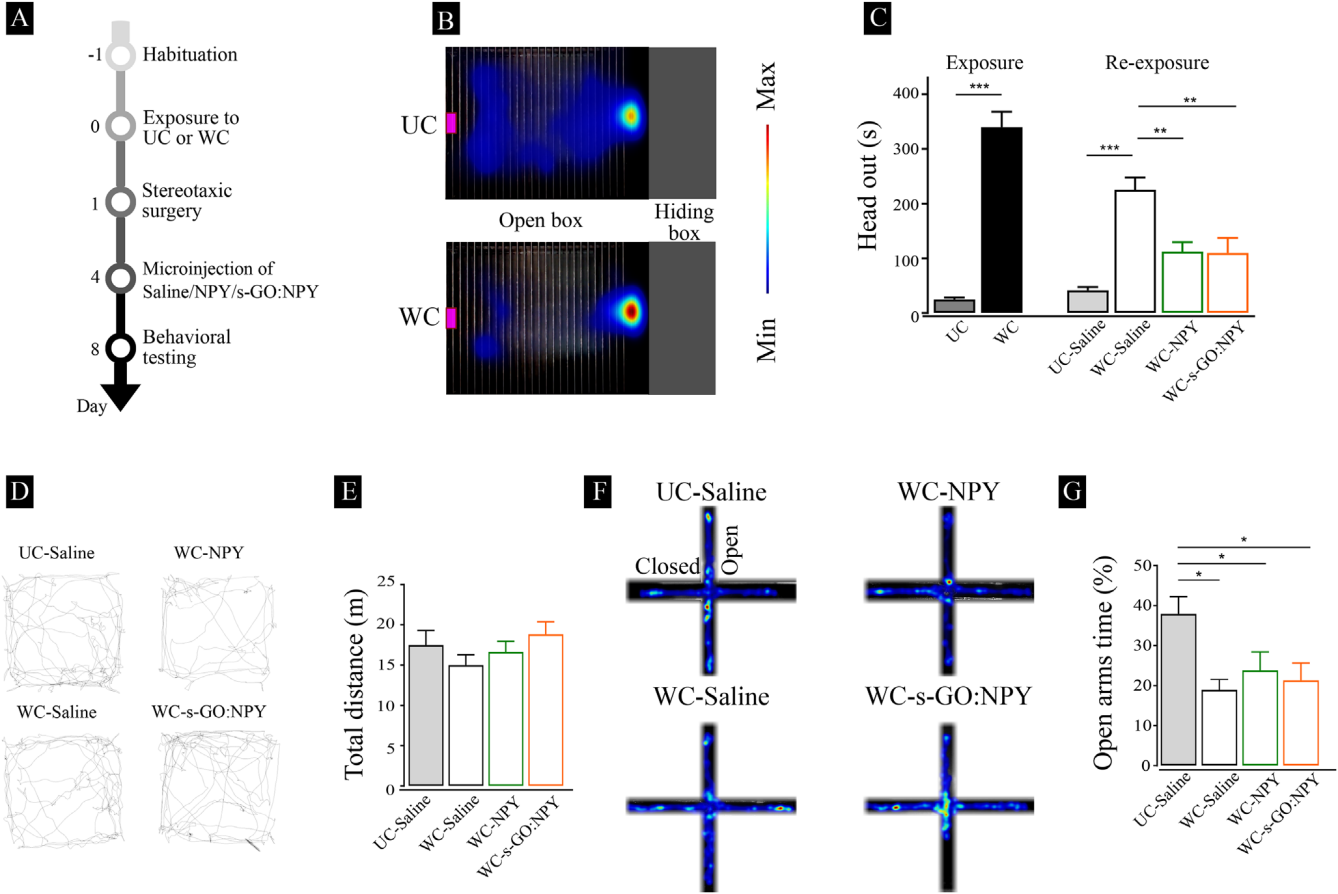
LA injected s‐GO:NPY impairs specifically fear memory related behaviors, but not anxiety related ones. (A) Experimental timeline. (B) Representative image of the open and hiding compartments of the avoidance box, showing cumulative heat maps of the head out behavior. (C) Bar plot summarizing the head‐out behavioral responses evoked by the exposure to UC or WC and by the re‐exposure to the context, in animals treated with saline, NPY or s‐GO:NPY. (D) Schematic representation of the OF illustrating the sample tracking in the apparatus by UC and WC groups treated with saline, NPY or with s‐GO:NPY. (E) Bar plot reporting the total distance travelled in the OF apparatus in UC and WC groups, treated with saline, NPY or with s‐GO:NPY. (F) Representation of the EPM, showing cumulative heat maps of the time spent in the arms of the EPM. (G) Bar plots depicting the time spent in the open arms of the EPM apparatus under the same treatments as described for UC and WC groups. *n* = 6 for each group. ^***^ *p* <0.001; ^**^ *p* <0.01; ^*^ *p* <0.05.

Next, in the PTSD model, we used the avoidance box, an apparatus to study contextual fear memory [49], and, upon context habituation, two independent groups of animals were exposed to a collar previously worn by a cat (WC) or to a control unworn collar (UC). 24 h after the first exposure to WC or UC, a guide cannula was stereotaxically implanted into the rat brain, targeting the LA to deliver locally, three days later, either NPY, s‐GO:NPY, or physiological saline solution, used as control, reaching the LA bregma coordinates between −2.64 and −3.96 mm (sketched in Figure 5A). Four days after the injections (i.e., 8 days after the odor exposure), the animals from the WC and UC groups were re‐exposed to the context in the avoidance box to measure the time spent in a “head out” defensive behavior [28], a hallmark of a contextual fear memory (Figure 5B) [50]. Figure 5C displays the increased head‐out defensive response in rats caused by either the cat odor exposure or the re‐exposure to the context 8 days later. In detail, cat odor exposure induced a significant innate fear response characterized by an increase in the head‐out behavioral response (t(10) = 11.05, *p* < 0.001) compared to the UC‐exposed group. Furthermore, the exposure to the predator odor also enhanced the head‐out response in the saline‐treated group during the re‐exposure to the context, evaluated 8 days later, compared to the saline‐treated group previously exposed to UC (F (3, 20) = 12.75, *p* < 0.001). However, in animals previously exposed to WC, both NPY and s‐GO:NPY injections decreased the emergence of the head‐out behavior (F (3, 20) = 12.75, *p* < 0.001), suggesting that the neuroactive NPY peptide, free or complexed to s‐GO, efficiently impaired the aversive memory behavior when delivered into the LA (Figure 5C).

We then tested locomotor activity by open field (OF, Figure 5D), and we did not detect changes in any group (F (3,20) = 1.14, *p* > 0.05; Figure 5E). Next, we tested by the elevated plus maze apparatus (EPM) [28] the anxiety‐related responses in all groups (Figure 5F). At day 8 after the predator odor exposed WC rats, injected with saline (t(10) = 2.94, *p* < 0.01)), NPY (t(10) = 2.23, *p* < 0.05) or s‐GO:NPY (t(10) = 2.7, *p* < 0.01) exhibited a statistically significant decrease in the time spent in the open arm of EPM, when compared to the UC‐saline treated group, a hallmark of an enhanced anxiety‐related behavior (*p* >0.05, Figure 5G). Indeed, long‐term anxiety‐related behaviors are supported by the activity of putative not expressing NPY receptors neuronal circuits [36], and the lack of specific targeting of these circuits by s‐GO:NPY complexes would explain why s‐GO:NPY, similarly to the peptide alone, did not rescue anxiety‐related behaviors.

Our experiments ruled out that the s‐GO:NPY induced behavioral changes were due to unspecific effect of the complex, since when this was administered after NPY Y1 receptors blockage via application of the antagonist BIBO 3304 (200 pmol) [51], as for free NPY, it was unable to rescue the contextual fear memory observed in saline injected WC animals (F (5, 29) = 4,899; F (5, 29) = 1,410, *p* > 0.05; Figure S7). A comparable lack of effect was observed when animals were injected with a complex carrying a scrambled NPY (s‐GO:NPYscr, same amino acids as in NPY but with a random sequence; *p* >0.05; Figures S7 and S8).

No behavioral modifications were identified in the EPM tests for animals pre‐treated with the antagonist BIBO 3304 before injecting s‐GO:NPY or in those injected with s‐GO:NPYscr (F (5, 29) = 1.737; *p* >0.05, Figure S7).″

## 3 Discussion

The main finding of this work is that peptide NPY, when non‐covalently complexed to s‐GO in a flat nanosheet‐based platform, retains its ability to bind and activate Y receptors, thus allowing, when intracranially administered into living brain tissue in vivo, modulation of specific neuronal pathways. We designed, through the non‐covalent adsorption strategy, a drug delivery system, in which medical‐grade s‐GO [23], previously found to be biocompatible for the CNS in vitro [24, 26] and in vivo [25, 27, 28, 29], was used as a platform to load NPY, a neuromodulator of glutamatergic synapses [30, 31, 32]. In dissociated hippocampal cultures, a standard in vitro model for testing vectorized drug delivery systems [52, 53, 54], that express NPY receptor‐positive neurons [37], we showed s‐GO:NPY Y1 and Y2 receptor‐mediated synaptic modulation, leading to a reversible downregulation of glutamatergic transmission, comparable to that of free NPY.

As amygdala synaptic potentiation of glutamatergic synapses has been described in contextual fear memory and PTSD like processes [28, 29, 34], we induced cLTP in dissociated amygdala cultures to test the impact of s‐GO:NPY on glutamatergic synapses potentiation. As in the case of free NPY, when s‐GO:NPY was co‐applied with the cLTP induction stimulus, no increment in the efficacy of glutamatergic neurotransmission was observed. We propose that the peptide, alone or complexed, binds to NPY receptors, expressed in these cultures, and by tuning pre‐synaptic glutamate release [55, 56], blocks LTP expression.

We used a rat model of contextual fear memory and PTSD, in which the exposure to a cue (i.e., a predator odor) leads to a dysfunctional long‐term plasticity of LA glutamatergic neurons [28], responsible for the emergence of long‐lasting fear and anxiety responses [44, 45]. Two distinct neuronal pathways have been proposed as the neuronal substrates of these distinct behaviors, departing from the LA and innervating the PAG or the VMH, respectively [46, 48, 57]. A previous study suggested that these pathways could be neurochemically identified by the differential expression of NPY receptors, with the LA‐vlPAG pathway expressing NPY (mainly Y1) receptors and the LA‐VMH not [36].

By confocal microscopy, we confirmed this hypothesis by retrogradely labeling LA neurons innervating the two regions [58] and showing NPY Y1 receptor‐positive neurons only in LA neurons innervating vlPAG, and thus responsible for fear memory‐related behaviors.

Our behavioral observations confirmed that NPY delivered through an s‐GO‐based drug delivery system affected only the LA‐vlPAG pathway. In fact, s‐GO:NPY injected WC animals, consistently with those treated with an equivalent dose of free NPY, presented a diminished head‐out behavior expression, indicative of reduced contextual fear memory response [28].

The specificity of NPY (complex or not) effects was strengthened by our EPM measures of long‐term anxiety‐related behaviors, and by OF characterization of locomotor activity, which were not affected by the peptide, as previously reported for NPY alone [36]. s‐GO:NPY drug delivery system selectively targeted the contextual fear memory related Y1 receptor expressing LA‐vlPAG neuronal pathway, but not the anxiety related Y1 receptor negative LA‐VHM one, indeed when co‐injecting Y1 receptor antagonist (BIBO 3304, 200 pmol) [51] fear memory responses were not modulated by s‐GO:NPY or uncomplexed NPY, as when using s‐GO:NPYscr, which prevented the binding of the neuroactive peptide to its receptor [59, 60].

Our findings, showing that, in agreement with a previous work [61], intra‐amygdala administration of NPY reduced contextual fear memory, while long‐term anxiety responses were unaffected, consolidate the hypothesis that stress‐related behaviors are supported by multiple distinct neural pathways that are differentially modulated, e.g. via selective expression of neuropeptides receptors [36]. These results suggest that the functional diversity of these pathways could be exploited for targeted pharmacological interventions.

In conclusion, nanosheet‐based transport systems onto which peptides can be adsorbed non‐covalently were able to target in vivo CNS cells/pathways expressing specific receptors. Although pristine s‐GO was previously reported to modulate glutamatergic presynaptic release per se [27, 28, 29], when complexed with large molecules this nanomaterial apparently loses such biological activity, strengthening its potential as a drug delivery platform for the CNS. Indeed, this is in accordance with what reported in a preliminary study where GO was covalently bound to the peptide [37]. This hypothesis is further supported by the observed inhibition of s‐GO:NPY when in the presence of NPY receptor antagonists, thus excluding any potential activity of the complex due to the s‐GO per se.

Our current experiments cannot discriminate between the mechanisms mediating NPY delivery, namely if NPY is released or presented by s‐GO:NPY. Although we cannot exclude the possibility of NPY release from the complexes, our current results reporting the absence of pristine s‐GO effects [28, 29] argue against NPY de‐complexing leading to significant amounts of free pristine s‐GO. Notably, the lack of s‐GO:NPY effects on the LA–VMH pathway—contrary to what is observed with uncomplexed s‐GO [28, 29], together with the loss of biological activity when in the presence of NPY receptor antagonists or when using s‐GO:NPYscr, favour the hypothesis that NPY remains largely associated with s‐GO during its biological activity.

Our experiments in which we injected the compounds intracranially, did not show different kinetics in the effect of NPY or s‐GO:NPY. However, in the context of therapeutic approaches where less invasive administration routes (e.g. intravenous, intranasal) could be used, the complexation of peptides to s‐GO might succeed in improving biological barrier crossing and translocation, with concomitant preservation of the bioavailability and biostability of peptides [62]. In addition, the versatile chemistry of s‐GO paves the way toward the design of drug delivery systems carrying multiple molecules, aimed to develop complex therapeutic/theranostic systems to be delivered to selective CNS targets.

## 4 Materials and Methods

### 4.1 Preparation and Characterization of Non‐Covalent s‐GO:NPY Complexes

#### 4.1.1 Reagents

Endotoxin‐free s‐GO was synthesized by our research group using the modified Hummers' method previously described (Rodrigues et al., 2018). Neuropeptide Y, NPY (1‐36), and neuropeptide Y scrambled, NPYscr (1‐36), were purchased from GenScript. Water for injection, essential for both s‐GO and complex preparation, was obtained from Grifols (Spain). All other chemicals were acquired from Sigma–Aldrich (Merck, Spain), unless otherwise specified. Amicon Ultra‐4 100 kDa MWCO Centrifugal Filter units were purchased from Merck Millipore (UFC810024).

#### 4.1.2 s‐GO:NPY Complex Preparation

Initially, NPY powder was reconstituted in water for injection at 1 mg/mL stock solution. The s‐GO sheets in water were adjusted to neutral pH using 0.1 m sodium hydroxide. We achieved the surface association of NPY to s‐GO through gentle mixing at room temperature for 30 min, in water for injection at varying s‐GO:NPY mass ratios, corresponding to 10:10, 10:4, 10:2, and 10:1, at the s‐GO initial concentration of 1 mg/mL. For control experiments, NPY was diluted to a final concentration of 400 µg/mL. s‐GO control at 1 mg/mL underwent the same preparation protocol, excluding the addition of NPY. For the purification of s‐GO:NPY and consequent removal of unbound NPY, 100 kDa Amicon ultra centrifugal filter units were employed. Specifically, the 10:4 mass ratio complex was subjected to four centrifugation cycles at 4000 g for 10 min at 20°C. Following each cycle, the filtrate (bottom fraction) containing unbound NPY was collected, and the volume of the purified fraction (retained above the membrane) was restored with water for the subsequent spin. An identical purification procedure was applied to the s‐GO control for comparative analysis. The loading efficiency of NPY onto the s‐GO sheets was indirectly determined by quantifying the concentration of unbound NPY in the collected filtrates using UV–vis spectrophotometry and HPLC.

#### 4.1.3 Scanning Electron Microscopy

Twenty µL of samples at s‐GO concentrations of 0.1 mg/mL were drop‐cast on grids with Ultrathin C on the Lacey C film (Ted Pella), and the excess of the droplet was removed by blotting. The procedure was repeated 5 times, and the samples were dried overnight at room temperature. SEM images were recorded at the ICN2 Electron Microscopy Unit with a Magellan 400L field emission microscope (Oxford Instruments) and an Everhart–Thornley detector for secondary electrons. The measurement conditions were 100 pA beam current and 20 kV acceleration voltage. The image processing was performed using ImageJ software (version 1.8.0).

#### 4.1.4 Atomic Force Microscopy

The samples were prepared by covering a cleaved mica surface (Ted Pella) with 20 µL of 0.01% poly‐L‐lysine solution (Sigma–Aldrich). After washing with water, 20 µL at the s‐GO concentration of 0.1 mg/mL were drop‐cast and washed again with water. For the measurements, the atomic force microscope Asylum MFP‐3D (Oxford Instruments) was used in air‐tapping mode. Silicon probes (Ted Pella) with a resonance frequency of 300 kHz and nominal force 40 N/m were used. AFM images of 5×5 µm were processed with Gwyddion software (version 2.57).

#### 4.1.5 Raman Spectroscopy

The samples for Raman analysis were prepared by drop‐casting 20 µL of 0.1 mg/mL of s‐GO onto glass coverslips, followed by overnight drying at room temperature. Raman measurements were recorded using a WITec confocal Raman microscope. A 633 nm laser with a 600 g/mm grating was used for excitation. Samples were irradiated with 1 mW for 10 s, and a 50× objective lens was selected for focusing. Prior to analysis, the spectra baseline was corrected. Data analysis was performed using PROJECT FOUR 4.1 software from WITec.

#### 4.1.6 X‐Ray Photoemission Spectroscopy

For XPS analyses, 20 µL of sample was drop‐cast multiple times onto a 5 × 5 mm silicon (Si) wafer (Ted Pella) to form a thin film. Measurements were acquired using a Phoibos 150 electron spectrometer (SPECS, GmbH) coupled with a hemispherical analyzer, operating under ultra‐high vacuum conditions and utilizing an Al Kα x‐ray source (hν = 1486.74 eV). These measurements were conducted at the ICN2 Photoemission Spectroscopy Facility. Charge effects were corrected by referencing the C1s line of adventitious carbon at 284.6 eV. Data analysis was performed using CasaXPS software.

#### 4.1.7 X‐Ray Diffraction

The samples were prepared by drop‐casting 200 µL of the sample dispersion onto a silicon (Si) holder, followed by drying in an oven at 50°C. The diffraction spectra were collected within a 2θ scan range from 5° to 60° using a Malvern PANalytical X'Pert Pro MPD diffractometer. Measurements, performed at the ICN2 XRD Facility, utilized a ceramic x‐ray tube with a Cu Kα anode (λ = 1.540 Å) as the x‐ray source and an X'Celerator solid‐state detector. All spectra were analyzed using X'Pert HighScore software (version 2.2c (2.2.3)).

#### 4.1.8 Dissociated Neuronal Cultures

All procedures were done in agreement with the Italian law (decree 26/14) and the EU guidelines (2007/526/CE and 2010/63/UE). The animal use was authorized by the Italian Ministry of Health (n. 689/2017‐PR, n. 22DAB.N.1Z8 and n. 22DAB.N.1WO) and approved by the local veterinary authorities and by the institutional (SISSA) ethical committee.

Dissociated hippocampal cultures were prepared from 2 to 3 days postnatal (P2−P3) Wistar rats as reported in [63] with minor modifications. After hippocampus isolation, cells were enzymatically and mechanically dissociated, then seeded on poly‐L‐ornithine‐coated glass coverslips (24 × 12 mm^2^, Kindler, EU) at a density of 250 000 cells/mL. Neuronal cultures were maintained in stable conditions (37°C, 5% CO_2_) in Neurobasal A medium (Gibco) added with 2% B27 Supplement (Life Technologies), 10 mm Glutamax (Gibco) and 500 nm gentamycin. After two days, the culture medium was replaced with one containing 1B‐arabinofuranosilcitosina (Ara‐C, 5 µm), to prevent glial over‐proliferation, and then changed every three days.

Dissociated amygdala cultures were obtained from P7‐10 Wistar rats and prepared as previously described [26] with slight modifications. In brief, rat brains were quickly removed from the skull and placed in fresh ice‐cold artificial cerebrospinal fluid (ACSF) containing (in mm): 124 NaCl, 24 NaHCO_3_, 13 glucose, 5 HEPES, 2.5 KCl, 2 CaCl_2_, 2 MgSO_4_, and 1,2 NaPO_4_H_2_ with a pH of 7.3–7.4 when saturated with 95% O_2_ and 5% CO_2_. Eight hundred µm thick coronal brain sections were cut using a vibratome (LeicaVT1000S), and under a dissecting microscope (Olympus SZ40), the regions containing the amygdaloid complex was visually identified following defined anatomical coordinates: Bregma −1.8, −2.4, and 2.8 mm [64]. Using a biopsy punch with a diameter of 1 mm (Kai Medical, Japan), the amygdala tissue was collected to be enzymatically and mechanically dissociated following standard protocol [63]. Cells were seeded onto poly‐L‐ornithine‐coated glass coverslips at a density of 1000 cells/mm^2^ and maintained in controlled conditions (at 37°C, 5% CO_2_) in Neurobasal A Medium (Gibco) containing B27 supplement (Thermofisher).

#### 4.1.9 Electrophysiology

Single cell patch clamp recordings of synaptic activity were obtained from both dissociated hippocampal and amygdala cultures after 8–15 days of differentiation in vitro. Voltage‐clamp whole‐cell recordings were performed at RT using glass micropipettes with a resistance of 4–7 MΩ once filled with the following intracellular saline solution (in mm): 120 K gluconate, 20 KCl, 10 HEPES, 10 EGTA, 2 MgCl_2_, 2 Na_2_ATP (pH 7.3, osmolarity adjusted to 300 mOsm). The standard extracellular solution contained (in mM) 150 NaCl, 4 KCl, 2 CaCl_2_, 1 MgCl_2_, 10 HEPES, 10 glucose (pH 7.4) and was continuously perfused at 2 mL/min. Cultures were mounted on a chamber and visualized with an inverted microscope (Eclipse TE‐200, Nikon, Japan). All data were collected by means of a Multiclamp 700A patch amplifier (Axon CNS, Molecular Devices) with a sampling rate of 10 kHz with the pClamp 10.6 acquisition software (Molecular Devices LLC, USA). Input resistance and cell capacitance were measured online with the membrane test feature of the pClamp software. Spontaneous activity was recorded in voltage‐clamp mode at a holding potential of −58 mV, not corrected for the liquid junction potential, which was −12 mV (calculated with the Clampex software; Molecular Devices, Sunnyvale, CA, USA). The stability of the recording was checked by repetitively monitoring the series resistance (<20 MΩ and not compensated) during the experiments, and cells showing 15% changes were excluded.

In experiments on dissociated hippocampal cultures, after monitoring baseline activity for at least 10 min, compounds were applied at concentrations of 1 µM for NPY and, 10 µg/mL and 1 µm, for s‐GO:NPY, respectively, through the inflow system for 5 min and then washed out for at least 7 min. As a control, the vehicle (extracellular solution) was applied without any compound. The effect of the treatments was quantified in the time interval of larger synaptic depression. This lasted ∼4 min and matched with the last 2 min of compound application (also considering that the delivery through the inflow system required ∼2 min) and the first 2 min of washout. The recovery after washout was evaluated in 5 min interval time between 7 and 12 min from the beginning of compound application (see experimental scheme in Figure 2A).

To induce cLTP in dissociated amygdala cultures, the protocol described in [28] was used (reproduced with slight modifications from [65]). Under voltage‐clamp mode, after recording spontaneous activity for 8 min as baseline, 50 µm of glutamate for 30 s was applied through the inflow system, while the membrane potential of the recorded cell was depolarized from −58 to +4 mV. s‐GO:NPY or NPY were applied through the perfusion system at a concentration of 10 µg/mL and 1 µm, respectively, for 5 min before the 30 s‐long lasting application of 50 µm of glutamate.

EPSCs were pharmacologically isolated upon application of 10 µm gabazine or identified in traces of spontaneous synaptic activity recorded in the absence of synaptic inhibitors on the basis of their kinetic properties. In this case, spontaneous PSCs were analyzed offline using the software AxoGraph X (Axograph Scientific), which exploits a detection algorithm based on a sliding template. Templates characterized by diverse decay times (т) were used to separate offline glutamate AMPA‐receptor mediated postsynaptic currents (EPSCs, т ∼3 ms) and those mediated by GABA_A_ ‐receptors (т ∼20 ms), as previously reported [28, 29].

For each recording, all collected events were averaged, and the peak amplitude was measured.

The effects of the compounds in the presence or absence of cLTP induction were monitored 18–24 min after the baseline collection through the measurement of EPSCs frequencies and amplitudes, in the absence of any drugs.

#### 4.1.10 Immunohistochemistry and Confocal Microscopy

Cultured amygdala neurons were fixed in PBS containing 4% paraformaldehyde for 20 min at RT. Cells were permeabilized with 1% Triton X‐100 for 30 min, blocked with 5% FBS in PBS for 30 min at RT, and incubated with primary antibodies for 60 min. The primary antibodies used were mouse polyclonal anti‐β‐tubulin III (Sigma, 1:250 dilution), guinea‐pig polyclonal anti GFAP (Alomone, 1:500 dilution), rabbit polyclonal anti‐NPY1 receptor (Abcam Plc, 1:500 dilution), and rabbit polyclonal anti‐NPY2 receptor (Thermofisher, dilution 1:2000). After the primary incubation and PBS washes, neurons were incubated for 60 min with the secondary antibodies AlexaFluor 488 goat anti‐rabbit (Invitrogen, dilution 1:500), AlexaFluor 594 goat anti‐mouse (Invitrogen, dilution 1:500), AlexaFluor 647 goat anti‐guinea pig (Invitrogen, dilution 1:500). Finally, cells were mounted on 1 mm thick glass coverslips using the Fluoromount mounting medium (Sigma–Aldrich). Images were acquired from randomly selected fields (70.64 µm × 70.64 µm) using a Nikon C2 Confocal, equipped with Ar/Kr, He/Ne, and UV lasers. Images were acquired with a 60× (1.4 NA) oil‐objective (using oil mounting medium, 1.515 refractive index).

#### 4.1.11 Experimental Design for In Vivo Procedure

Behavioral experimental procedures were carried out in accordance with the Italian law (decree 26/14) and the EU guidelines (2007/526/CE and 2010/63/UE) and were approved by the Italian Ministry of Health (n. 22DAB.11). All experimental procedures were planned to minimize the number of animals used and their suffering. Male adult Wistar rats weighed 230–300 g (*n* = 48) were used to perform the in vivo experiments. Rats received food and water at libitum. The enclosure was maintained at 21°C ± 2°C on a light‐dark cycle (lights on from 7 p.m. to 7 a.m.). Behavior testing was performed as described previously [28]. Briefly, aversive memory‐related responses were evaluated in the avoidance box apparatus, which comprises a rectangular arena (40 × 26 × 36 cm) and two compartments. One of them is a black acrylic‐plexiglass wall covered with a transparent plexiglass lid. At one side of the arena, an alligator clip fixed in the wall is positioned 4 cm above the floor. The second compartment is a smaller box (20 × 26 × 22 cm) covered with a black plexiglass lid, named hide box, positioned in the opposite direction of the rectangular arena. Both compartments were separated by a small 6 × 6 cm square hole allowing free access to both chambers. Animals were placed inside the smaller box with free access to the arena to habituate to the apparatus for 10 min during 3 consecutive days. On the fourth day, the duration of the following defensive behavior was recorded: head out (when the rat scanning the arena from a protected position, measured as poking of the head, or of head and shoulders, outside of the hide box but with the bulk of the rat body inside the smaller compartment). After, rats were divided in two groups (*n* = 6 per group), exposed to either a piece (2 cm) of an unworn collar (UC), without any predator odor or a piece of the collar previously worn by a cat, named worn collar (WC). All collars were previously worn by an encaged cat. Eight days later, rats were re‐exposed (10 min) to the context, arena without the cat collar to evaluate the aversive memory related to the conditioned fear. Head‐out behavioral response was analyzed during the re‐exposure to the context. Right away, animals were placed in the elevated plus maze (EPM) to evaluate the long‐term anxiety‐related behavior. EPMs consisted of four arms (50 × 10 × 40 cm), two closed arms (with 40 cm high walls) and two open arms 127 (without walls) connected by a central square (10 × 10 cm). The maze was elevated 50 cm from the ground. Rats (*n* = 6 per group) were placed in the closed arm with free access to explore the apparatus for 5 min. Time spent in the open zone was evaluated. Exploratory and locomotor activities of rats (*n* = 6 per group) were also evaluated in the open field (OF) apparatus, which consists of a square arena with the 60 × 60 × 40 cm black plexiglass walls and floor. Total distance moved (cm) in the OF were analyzed after the EPM testing. All behavioral trials were performed between 8 a.m. and 1 p.m. under low luminosity (12 lx) and videorecorded for off‐line analysis. The XPloRat software [66] and EthoVision XT software were used to score the behaviors. Next day, animals were submitted to stereotaxic surgery as described previously [67]. Briefly, animals received an intraperitoneal injection of ketamine (Ketamine Imalgene, Merial Laboratories) and xylazine (Sedaxylan, Dechra Veterinary Products) at 92 and 10 mg/kg body weight, respectively, and their heads were fixed in a stereotaxic frame. A stainless‐steel guide cannula (outer diameter, 0.6 mm, and inner diameter, 0.4 mm) was implanted in the diencephalon directed to the LA. The skull was positioned horizontally between bregma and lambda. The guide cannula was vertically introduced into the brain using bregma as the reference and the following coordinates: A.P.−3.4 mm, M.L.−5.2 mm, and D.V.−7 mm, according to Paxinos G. and Watson C.R., 2007. At the end of the surgery, the acrylic resin and one stainless steel screws were used to fix the guide cannula in the skull. In order to protect the guide cannula from obstruction a stainless‐steel wire was used to seal it. After surgery, analgesic and antibiotic medications were administrated. Three days later, rats were gently wrapped in a cloth and held while they received a random and single treatment of either NPY (5 µM), s‐GO:NPY (50 µg/mL and 5 µm, respectively), or saline (standard extracellular solution; composition described below) delivered by a needle (0.3 mm of outer diameter) linked to a micro‐syringe (Hamilton) through a polyethylene tube into LA. An independent group of rats received injections of combined treatments as follow: BIBO 3304 + saline, BIBO 3304 + s‐GO:NPY, scrambled NPY (s‐GO:NPYscr) + saline or s‐GO:NPYscr + s‐GO:NPY. Injections were performed at 10 min intervals. The microneedle was inserted through the guide‐cannula until it reached the LA (2 mm below the guide‐cannula). Only the rats where the needle tip reached LA were included in the study. Four days later or eight days after the exposure to the collar, animals were submitted to behavioral testing.

#### 4.1.12 Labelling of LA Pathways: Neurotracer Application and Immunostaining Techniques

A needle (0.3 mm of outer diameter) linked to a micro‐syringe (Hamilton) through a polyethylene tube was filled with the Fast Blue fluorescent tracer (Polysiences) at 2% dissolved in 0.01 m phosphate‐buffered saline (PBS), pH 7.4. Animals (*n* = 2) were anesthetized using ketamine at 92 mg/kg and xylazine at 9.2 mg/kg and fixed in a stereotaxic frame. The upper incisor bar was set at 3.3 mm below the interaural line so that the skull was horizontal between bregma and lambda. The needle filled with the neurotracer solution was vertically introduced into either vlPAG or VMH using the following coordinates, using bregma as the reference: A.P. −7.2 or −2.4 mm, M.L. −0.6 or −0.6 mm, and D.V. −5.6 mm or −9.4, respectively, according to [68]. Fast blue was injected into the brain structures in a volume of 0.5 µL (0.1 µL per minute). The needle was maintained in place for 1 min after the injection was complete to avoid leakage of the neurotracer. It was then withdrawn from the brain, and the bone was subsequently closed using dental cement. Fourteen days after the injection of the neurotracer, the animals were anaesthetized with ketamine and xylazine, as described previously, and perfused via the left cardiac ventricle. The blood was washed out using 200 mL of buffered saline followed by 200 mL of 4% (w/v) paraformaldehyde in 0.1 m PBS, pH 7.3, at a pressure of 50 mm Hg. Then the rat's brain was extracted, following cryosectioning of the tissue, slices were mounted on positively charged glass slides and washed once with PBS (0.1 m).

In order to identify the fiber projecting to the LA expressing the Y1 NPY receptor, slices were further processed for immunofluorescence with primary antibody rabbit anti‐NPY1 receptor (1:500; Biorbyt) in 5% FBS in PBS incubated overnight at 4°C. Following washing in PBS, sections were incubated for 2 h at room temperature in secondary antibodies (goat anti‐rabbit Fluor 594, 1:400, Invitrogen) in 5% FBS in PBS.

All sections were mounted with Fluoromount (Invitrogen) and visualized using the confocal microscope (Nikon A1 R) acquired with and 4x large image, 10×, 20×, and 40× objective (n.a. 1.2) using oil‐mounting medium and analyzed using *ImageJ* software, enabling qualitative assessments of the tracer labeling. Fast Blue neurotracer images were acquired using the 406 nm laser on confocal microscopy, visualizing the tracer in blue. However, using ImageJ software, projections from vlPAG were processed and visualized using a lookup table that rendered the images in green for better contrast and analysis.

#### 4.1.13 Data Analysis and Statistics

All values from samples subjected to the same experimental protocols were pooled together and expressed as mean ± s.e.m with *n* = animals or cells, unless otherwise indicated.

Data from independent groups of rats were checked for normality and homogeneity. Comparisons between independent variables were made with one‐way ANOVAs, followed by Tukey’ multiple comparison test and Student's unpaired one‐tailed or two‐tailed *t*‐test when appropriate.

For electrophysiological data, Shapiro–Wilk normality test was applied to evaluate the statistical distribution of the data sets. Statistically significant differences among data sets were made with one‐way ANOVA for repeated measures (Friedman test), corrected for multiple comparisons through Dunn's test, or with Wilcoxon test.

## Author Contributions

The manuscript was written through contributions of all authors. All authors have given approval to the final version of the manuscript. EP performed all in vitro experiments and analysis; AFB and RC performed all in vivo experiments and analysis; NL and KK performed and designed the s‐GO experimental strategy; NL and LMA synthesized and characterized the s‐GO batch; LMA, TB and GG prepared and characterized the s‐GO complexes, AB designed experiments with scrambled NPY; KK, GC and LB conceived the study; GC and LB designed the experimental plan, interpreted the results and wrote the manuscript.

## Conflicts of Interest

The authors declare no conflicts of interest.

## Supporting information




**Supporting File**: advs76608‐sup‐0001‐SuppMat.pdf.

## Data Availability

The data that support the findings of this study are available from the corresponding author upon reasonable request.
