# Supplementary material for: Neuropeptide Y—Graphene Oxide Complexes Inhibit Amygdala NPY‐Receptor Expressing Glutamatergic Pathways and Selectively Remove Aversive Memory In Vivo
Source: Adv Sci (Weinh). 2026 Jul 31;13(56):e76608. doi: 10.1002/advs.76608 (PMC13426394; doi:10.1002/advs.76608)
Supplement: Supplementary file 1 [file ADVS-13-e76608-s001.pdf]

SUPPORTING INFORMATION

Neuropeptide Y - graphene oxide complexes inhibit amygdala NPY-receptor expressing glutamatergic pathways and selectively remove aversive memory *in vivo*

Elisa Pati[1], Audrey Franceschi Biagioni[1], Raffaele Casani[1], Luis M. Arellano[2], Tommaso Battisti[2], Gloria Garcia-Ortega[2,3], Neus Lozano[2,3], Alberto Bianco[4], Kostas Kostarelos[2,3,5,6]*, Laura Ballerini[1,#]* and Giada Cellot[1,7,#]*

[1]*International School for Advanced Studies (SISSA/ISAS), 34136 Trieste, Italy*
[2]*Nanomedicine Lab, Catalan Institute of Nanoscience and Nanotechnology (ICN2), CSIC and BIST, Campus UAB, 08193 Barcelona, Spain*
[3]*Institute of Neuroscience, Universitat Autònoma de Barcelona, 08913 Barcelona, Spain*
[4]*CNRS, Immunology, Immunopathology and Therapeutic Chemistry, UPR3572, University of Strasbourg, ISIS, 67000 Strasbourg, France.*
[5]*Centre for Nanotechnology in Medicine, Faculty of Biology, Medicine & Health, The University of Manchester, Manchester, UK*
[6]*ICREA, Passeig de Lluís Companys 23, 08010 Barcelona, Spain*
[7]*University of Trieste, Department of life sciences, 34127 Trieste, Italy*

[#]equal contribution
[*]corresponding authors

SUPPLEMENTARY EXPERIMENTAL MATERIALS AND METHODS

Characterization of non-covalent s-GO:NPY complexes

UV-Vis spectrophotometry: Absorbance measurements were performed on s-GO control and s-GO:NPY complexes, prepared by dilution in water to concentrations of 2.5–20 µg/mL for s-GO. Spectra were acquired using an Evolution 201 UV-Vis spectrophotometer (Thermo Scientific). Calibration curve for s-GO control (at 230 nm) was established within the aforementioned concentration ranges, demonstrating excellent linearity (R^2 = 0.9996). Unbound NPY concentrations were calculated via interpolation from the established NPY calibration curve by HPLC. All spectral data underwent analysis utilizing Origin software (version b9.5.0.193).

High-Performance Liquid Chromatography: NPY chromatograms were acquired using a PerkinElmer Flexar HPLC system equipped with a multiwavelength UV-Vis photodetector. Separation was achieved on a C18 Hypersil BDS column (4.6×150mm, 5 µm, ThermoFisher). The mobile phase consisted of 0.1% trifluoroacetic acid (TFA) in water (Mobile Phase A) and 0.1% TFA in acetonitrile (Mobile Phase B), utilized in an isocratic elution at a 50:50 (v/v) ratio. A constant flow rate of 1 mL/min was maintained, with an injection volume of 10 µL. NPY was detected at 254 nm, exhibiting a retention time of approximately 5.6 min. The calibration curve for NPY showed linearity from 5 to 100 µg/mL, with a correlation coefficient (R^2) of 0.999.

Stability evaluation of the s-GO:NPY complex: To assess the long-term colloidal stability of the s-GO:NPY complex, purified suspensions of both the complex and the s-GO control were stored at room temperature under dark conditions. At different time points, the size, polydispersity index and zeta potential were evaluated by DLS, and the extent of NPY detachment from the s-GO surface was investigated by HPLC.

Direct quantification of bound NPY: To directly quantify NPY bound to the s-GO surface, purified s-GO samples were subjected to forced peptide desorption using SDS/NuPAGE combined with heating at 80°C. These conditions promote the dissociation of NPY from the s-GO surface. Following detachment, the released NPY was separated from the s-GO by centrifugation and quantified by HPLC.

Dynamic Light Scattering and zeta potential measurements: The size and zeta potential were measured using a Zetasizer Nano ZS (Malvern Instruments). One mL of each sample at s-GO concentration of 20 µg/mL were prepared and loaded into disposable capillary cells. Water dispersant settings for viscosity and refractive index were selected, and each sample was measured in triplicate at room temperature. Data was analyzed with Zetasizer software (version 7.12) and are presented as mean ± standard deviation unless otherwise specified.

NPY detachment from the s-GO sheets: NPY detachment experiments for the purified complex were performed at specific time points. The experiment involved centrifuging the samples four times at 4000 g for 10 minutes at 20 °C, utilizing 100 kDa Amicon Ultra Centrifugal Filter devices. Similar to the initial purification process, the amount of NPY collected in each filtrate was quantified using UV-Vis spectrophotometry and HPLC.

TNF-α expression test: TET was performed to detect endotoxin content in s-GO control and s-GO:NPY upon exposure of human bone marrow-derived macrophages, as previously described (Mukherjee SP et al, 2016).

SUPPLEMENTARY FIGURES

A

Graphene oxide nanosheets (s-GO) properties in a water suspension

Physicochemical properties	Technique	Results*
Lateral dimension	Optical microscopy	Non detectable (< 2μm)
	Scanning electron microscopy	50 - 950 nm (n = 946)
		95% < 450 nm
		Mean 144 nm
	Atomic force microscopy	10 - 890 nm (n = 2890)
		95% < 350 nm
		Mean 72 nm
Thickness	Atomic force microscopy	1 nm (1 layer)
Optical properties	UV-Vis spectrophotometry	ε_{232} (mL μg^{-1} cm^{-1})= 0.050
Degree of defects $(I_D/I_G)_{633nm}$	Raman spectroscopy	1.12 ± 0.02
Peak (2θ)	X-ray diffraction	12.36 °
Interlayer distance (nm)		0.71
Surface charge (ζ-Potential)	Electrophoretic mobility	- 47.2 ± 0.7 mV
Functional groups	Fourier transform infrared spectroscopy	ν(C-H): 2838 cm-1
		ν(C=O): 1735 cm-1
		ν(C=C): 1645 cm-1
		ν(O-H): 1423 cm-1
		ν(C-O): 1062 cm-1
Functionalization degree	Thermal gravimetric analysis	30-75°C: 7% (water)
		200-250°C: 30%
		250-950°C: 20%
		TOTAL 50%
Purity C+O (%)	X-ray photoelectron spectroscopy	98.1
Oxygen content (%)		29.6
Carbon content (%)		68.5
Nitrogen content (%)		1.1
Sulphur content (%)		0.9
C:O ratio		2.3

** Results are presented as mean values ± SD of at least 3 technical replicates. n-number in optical microscopy, scanning electron microscopy and atomic force microscopy indicate the number of individual GO sheets analysed.*

(i)

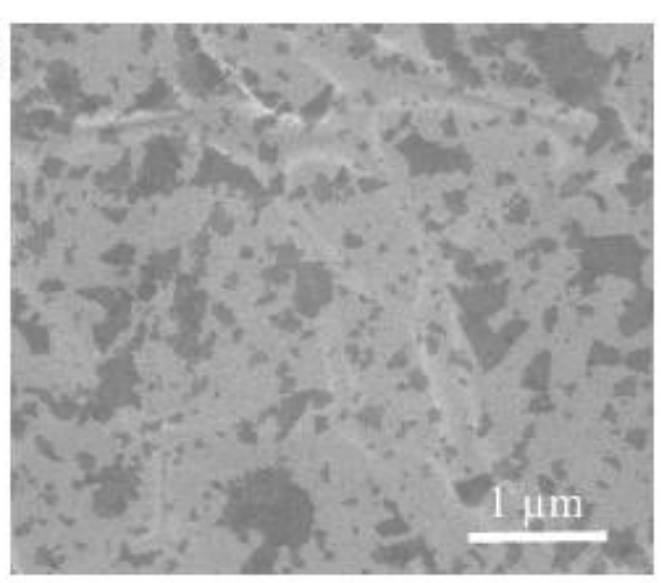

(ii)

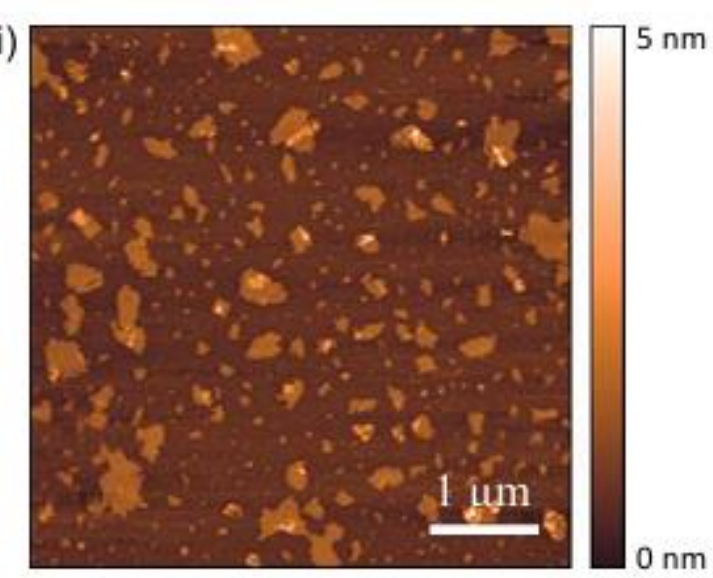

B

Neuropeptide Y (NPY)

NPY (1-36):

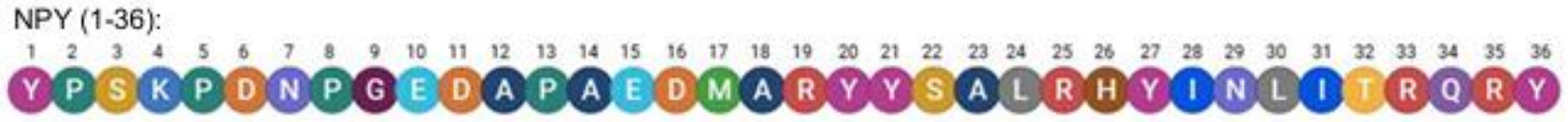

Molecular formula: $C_{189}H_{284}N_{54}O_{58}S_1$
Molecular weight: 4 272.7 g/mol
Purity: ≥ 95.0%

Supplementary Figure S1: Starting materials used to prepare s-GO:NPY. (A) Table with the main physicochemical properties of s-GO sheets in water with representative (i) SEM and (ii) AFM micrographs. (B) Amino acid sequence of NPY (1-36).

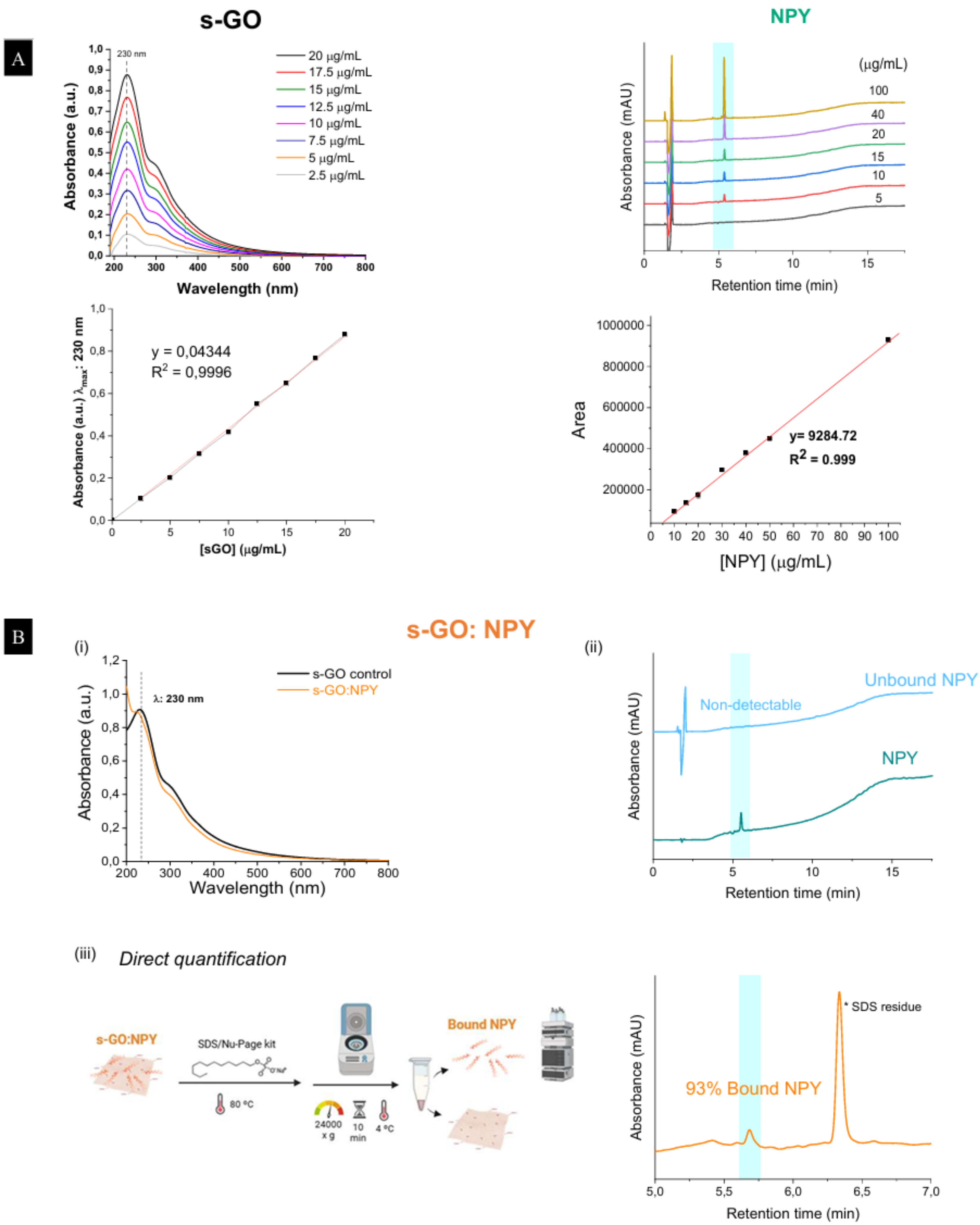

Supplementary Figure S2: Quantification of s-GO and NPY after the non-covalent complexation. (A) Calibration curve of s-GO control from 2.5 to 20 μg/mL using UV-Vis spectrophotometry, and of NPY from 5 to 100 μg/mL using HPLC. (B) Quantification of (i) s-GO and (ii) NPY after complexation using UV-Vis and HPLC, respectively, and (iii) schematic for the direct quantification of bound NPY in the s-GO:NPY complex using SDS/Nu Page at 80ºC, to detach the NPY associated to the s-GO, and further quantification by HPLC. Schematic was created with BioRender.com.

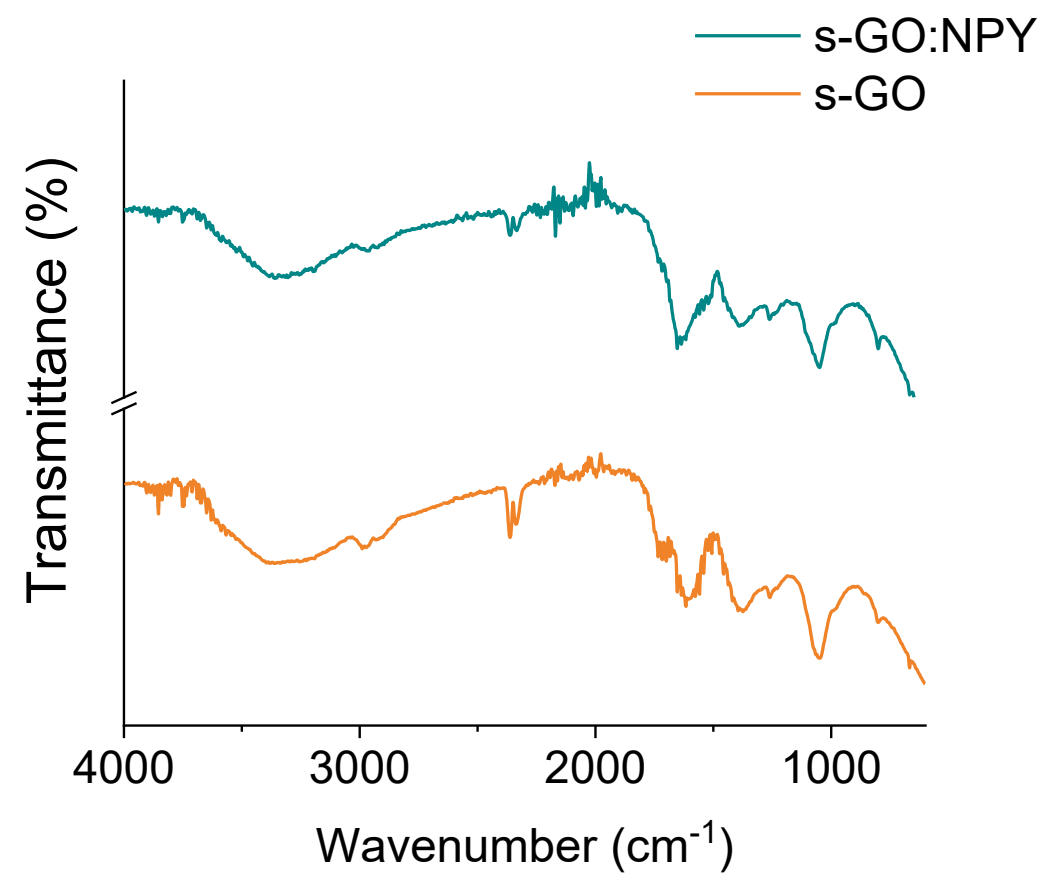

Supplementary Figure S3: FTIR-ATR of s-GO and s-GO:NPY.

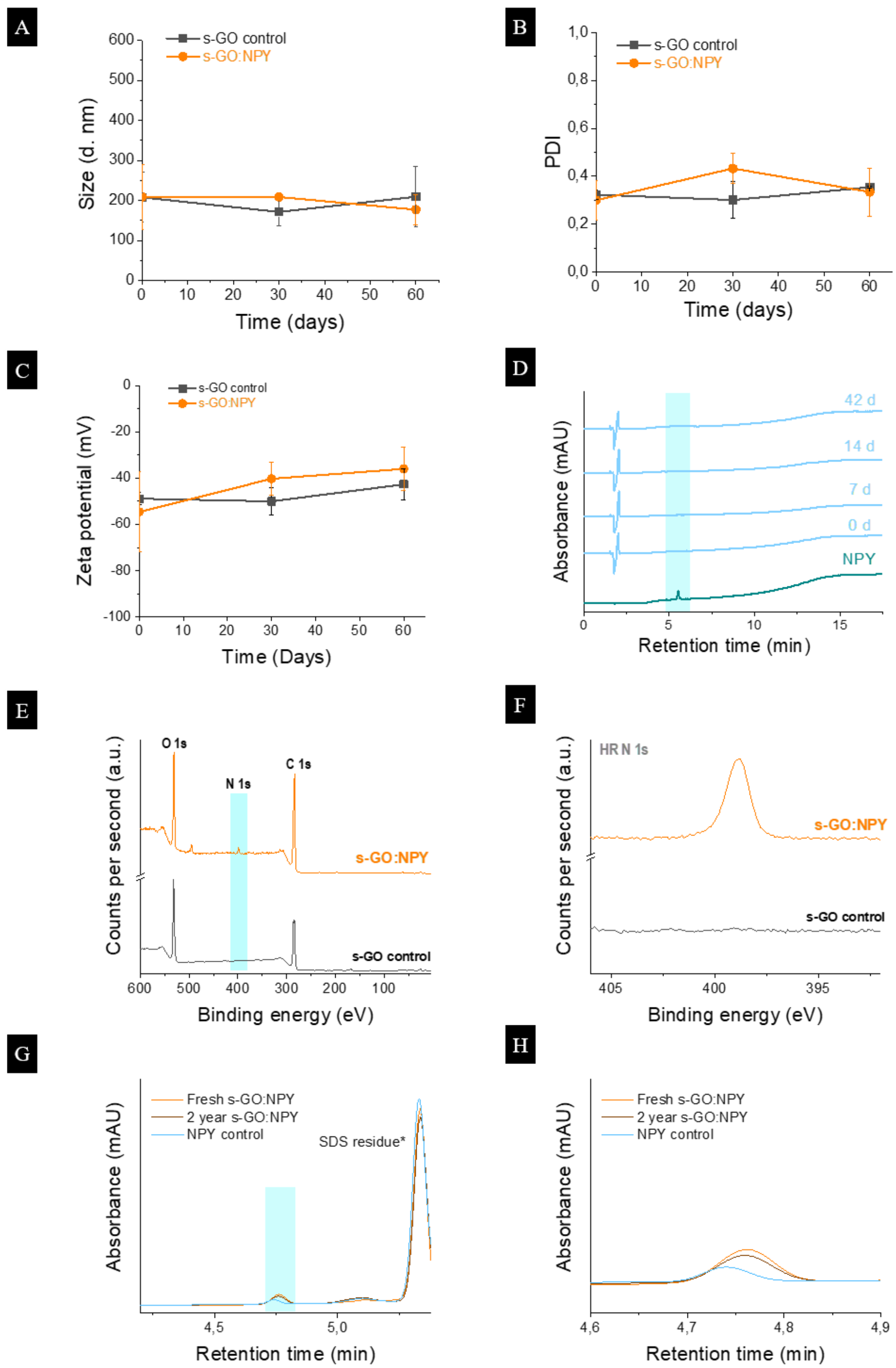

Supplementary Figure S4: Stability of s-GO control and s-GO:NPY overtime. (A) Hydrodynamic diameter, (B) polydispersity index, and (C) zeta potential of s-GO control and s-GO:NPY over 60 days (n=2). (D) Quantification of unbound NPY overtime with no evidence of NPY released. **(E)** XPS survey spectra of s-GO and s-GO:NPY after 30 days of aging, confirming the presence of nitrogen (N 1s), and **(F)** the corresponding high-resolution N 1s spectra. **(G)** HPLC analysis of NPY released from the s-GO surface, compared with a 2-year-aged NPY reference sample. **(H)** Corresponding chromatograms (inset), confirming the retention of NPY on s-GO and evidencing the exceptional stability of the s-GO assembly over time.

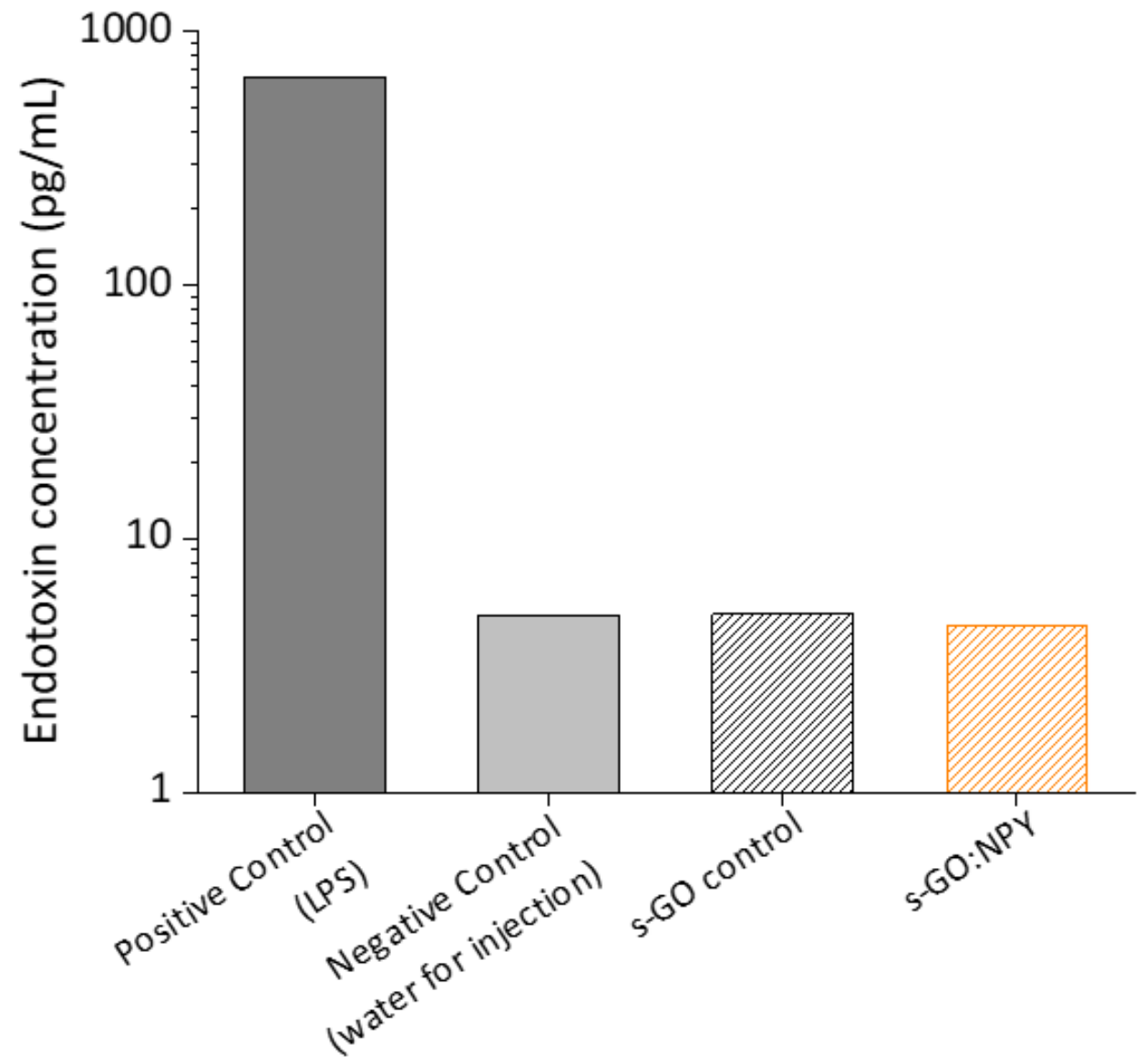

Supplementary Figure S5: TNF-α expression test for s-GO control and s-GO:NPY. The differences in TNF-α expression in the presence and absence of polymyxin B sulfate (10 μM) provided evidence of the absence of endotoxin content in both samples s-GO control and s-GO:NPY, as for the negative control.

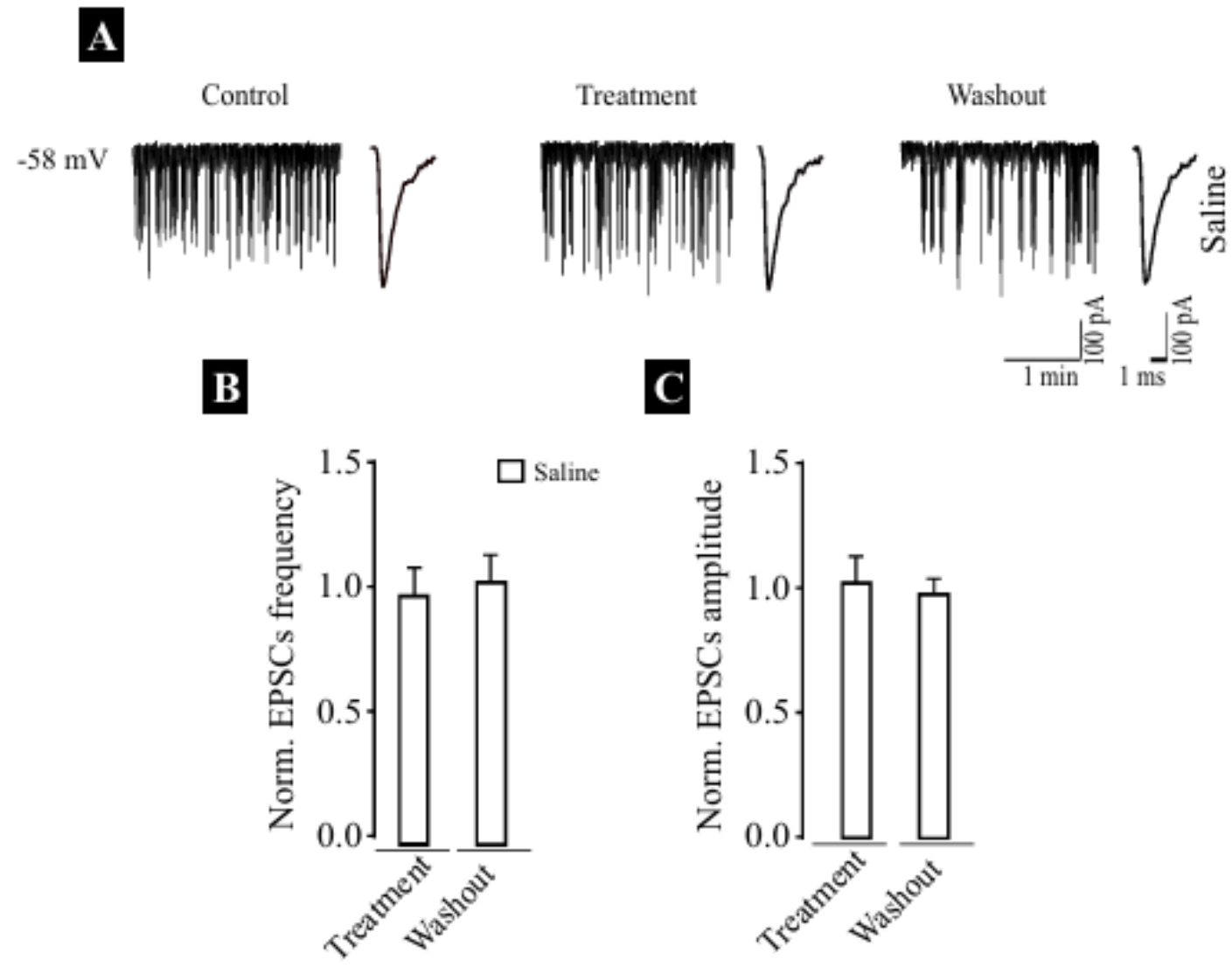

Figure S6: **EPSCs present stable frequency and amplitude upon saline treatment in dissociated hippocampal cultures.** (A) For saline treatment, exemplificative traces of recordings performed in the presence of 10 µM gabazine, showing no changes in glutamatergic synaptic activity. On the right of each trace, averaged EPSCs measured in the different phases of the experiment are reported. Bar plots of normalized EPSCs frequency (B, during treatment 0.94 ± 0.11 and during wash out 1.03 ± 0.11) and amplitude (C, during treatment 1.02 ± 0.08 and during wash out 0.95 ± 0.05, in n=6 saline treated cells) for the different experimental phases.

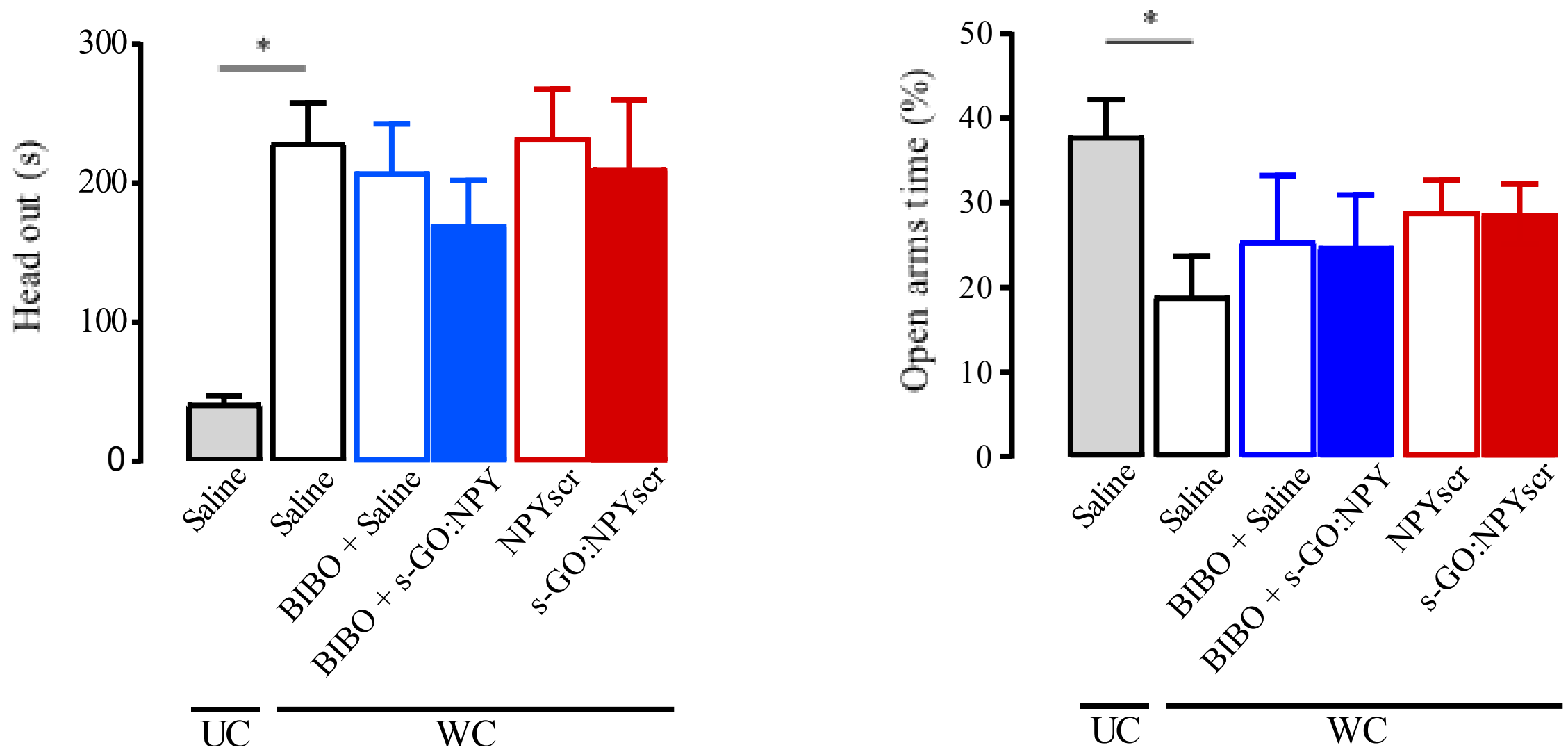

Supplementary Figure S7: **Behavioral responses modulation by s-GO:NPY depends on NPY receptor activation in the LA.** Bar plots summarizing (left) the head out behavior evoked by re-exposure to the context 8 days after the exposure to UC or WC collar, and (right) the time spent in the open arms of the EPM, in animals treated with saline, BIBO 3304 and saline, BIBO 3304 and s-GO:NPY, NPYscr or s-GO:NPYscr. n = 5-6 for group. *p<0.05.

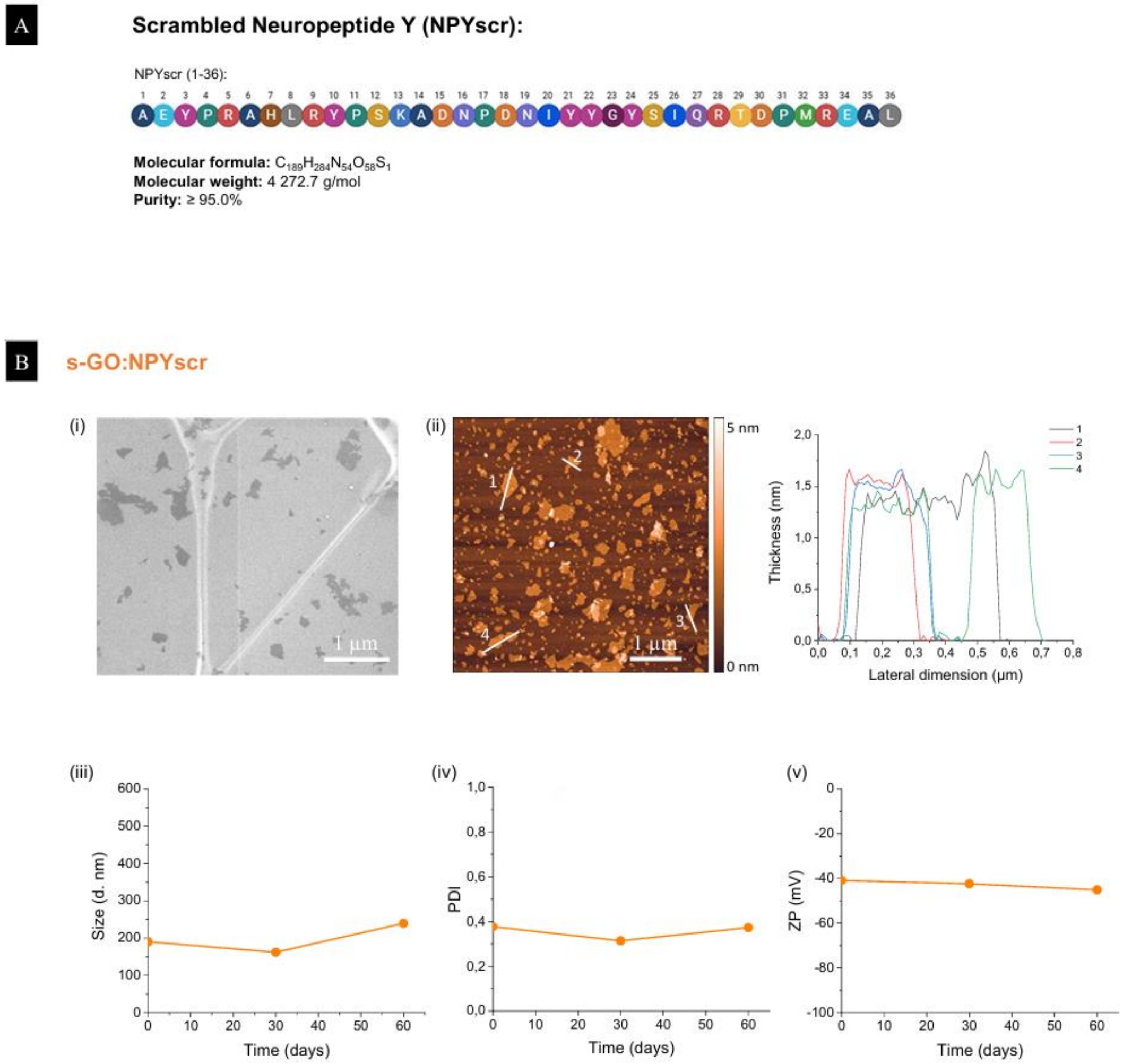

Supplementary Figure S8: Non-covalent complexation of s-GO with NPYscr. (A) Aminoacid sequence of the scrambled neuropeptide (NPYscr). (B) Morphological and colloidal characterization of s-GO:NPYscr: (i) SEM micrograph, (ii) AFM height image and cross-sections analysis. Stability overtime of s-GO control and s-GO:NPYscr: (iii) hydrodynamic diameter, (iv) polydispersity index, and (v) zeta potential measurements.

SUPPLEMENTARY TABLES

Table S1. Kinetic properties of EPSCs in dissociated hippocampal cultures upon treatment with saline, NPY or s-GO:NPY

	**Rise time (ms)**			**Decay time (ms)**			**cells**	**P value**
	control	treatment	washout	control	treatment	washout		
**Saline**	0.51±0.04	0.53±0.04	0.53±0.04	3.09±0.68	3.21±0.58	3.63±0.66	n=6	p>0.05
**NPY**	0.54±0.06	0.58±0.05	0.57±0.05	4.49±0.69	4.15±0.86	3.89±0.74	n=7	p>0.05
**sGO:NPY**	0.47±0.02	0.52±0.07	0.51±0.06	4.02±0.83	3.91±0.52	3.94±0.71	n=10	p>0.05

SUPPLEMENTARY REFERENCES

Mukherjee, S.P., Lozano, N., Kucki, M., Del Rio-Castillo, A.E., Newman, L., Vázquez, E., Kostarelos, K., Wick, P., Fadeel, B. Detection of Endotoxin Contamination of Graphene Based Materials Using the TNF-α Expression Test and Guidelines for Endotoxin-Free Graphene Oxide Production. PLoS One. 2016 Nov 23;11(11):e0166816. doi: 10.1371/journal.pone.0166816.
